# CRTAC1 enhances the chemosensitivity of non-small cell lung cancer to cisplatin by eliciting RyR-mediated calcium release and inhibiting Akt1 expression

**DOI:** 10.1038/s41419-023-06088-1

**Published:** 2023-08-26

**Authors:** Zihui Jin, Lingling Zhao, Yixin Chang, Rongjia Jin, Fangyu Hu, Shuang Wu, Zixuan Xue, Yimeng Ma, Chenglin Chen, Minghui Zheng, Yuanyuan Chang, Honglei Jin, Qipeng Xie, Chuanshu Huang, Haishan Huang

**Affiliations:** 1 Zhejiang Provincial Key Laboratory of Medical Genetics, Key Laboratory of Laboratory Medicine, Ministry of Education, School of Laboratory Medicine and Life Sciences, Wenzhou Medical University, 325035 Wenzhou, Zhejiang China; 2 Center for Molecular Diagnosis and Precision Medicine, and The Department of Clinical Laboratory, The First Affiliated Hospital of Nanchang University, 17 Yongwai Zhengjie, 330006 Nanchang, China; 3 Department of Laboratory Medicine, The Second Affiliated Hospital & Yuying Children’s Hospital of Wenzhou Medical University, 325035 Wenzhou, Zhejiang People’s Republic of China; 4 Oujiang Laboratory (Zhejiang Lab for Regenerative Medicine, Vision and Brain Health), 325035 Wenzhou, Zhejiang China

**Keywords:** Chemotherapy, Predictive markers

## Abstract

Sensitivity to platinum-based combination chemotherapy is associated with a favorable prognosis in patients with non-small cell lung cancer (NSCLC). Here, our results obtained from analyses of the Gene Expression Omnibus database of NSCLC patients showed that cartilage acidic protein 1 (CRTAC1) plays a role in the response to platinum-based chemotherapy. Overexpression of CRTAC1 increased sensitivity to cisplatin in vitro, whereas knockdown of CRTAC1 decreased chemosensitivity of NSCLC cells. In vivo mouse experiments showed that CRTAC1 overexpression increased the antitumor effects of cisplatin. CRTAC1 overexpression promoted NFAT transcriptional activation by increasing intracellular Ca^2+^ levels, thereby inducing its regulated STUB1 mRNA transcription and protein expression, accelerating Akt1 protein degradation and, in turn, enhancing cisplatin-induced apoptosis. Taken together, the present results indicate that CRTAC1 overexpression increases the chemosensitivity of NSCLC to cisplatin treatment by inducing Ca^2+^-dependent Akt1 degradation and apoptosis, suggesting the potential of CRTAC1 as a biomarker for predicting cisplatin chemosensitivity. Our results further reveal that modulating the expression of CRTAC1 could be a new strategy for increasing the efficacy of cisplatin in chemotherapy of NSCLC patients.

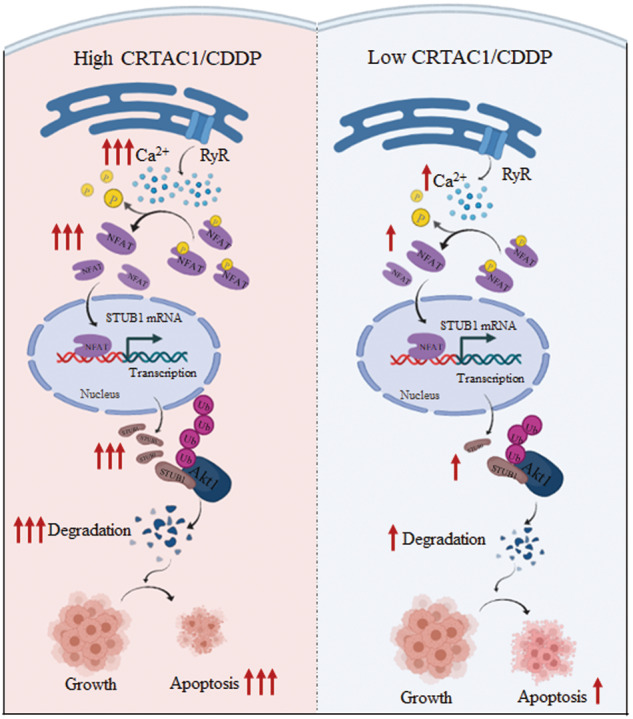

## Introduction

Lung cancer is the leading cause of cancer-related death in the world, and approximately 4/5 of lung cancers are non-small cell lung cancer (NSCLC) [1]. For many years, chemotherapy has been the mainstream treatment for NSCLC patients at different stages who require systemic therapy, and its use has increased the absolute 5-year survival rate of patients by 5–15% [2]. Chemotherapy will likely continue to be the cornerstone of the treatment of NSCLC in the future. It can be used as part of adjuvant chemotherapy for patients who undergo early surgical resection of NSCLC, as palliative treatment for patients with advanced metastatic NSCLC, and as bimodal or multimodal treatment for patients with locally advanced NSCLC throughout the course of the disease [3]. The chemotherapy regimens recommended by the National Comprehensive Cancer Network guidelines include platinum-containing dual-agent chemotherapy as the main first-line treatment, while cisplatin (Cis-diamminedichloride platinum, CDDP) is the most commonly used standard first-line chemotherapy drug [4]. However, cytotoxic chemotherapy is not always effective. Since the approval of cisplatin for clinical application in 1978, there have been few novel chemotherapy drugs and no further breakthrough in the efficacy of chemotherapy for NSCLC [5]. The development of chemotherapy resistance and adverse reactions in individual cases are important limiting factors. Despite this, 85–90% of patients still receive platinum-based chemotherapy [6]. Thus, identifying novel biomarkers for the prediction of platinum-sensitive patients should be highly significant in the chemotherapy of NSCLC patients.

The cartilage acidic protein 1 (CRTAC1) gene encodes a glycosylated extracellular matrix protein [7] with a tissue-specific expression that is highly expressed in normal lung tissue. Lung function studies show that CRTAC1 in bronchoalveolar lavage fluid and plasma serves as a biomarker of the health of type II alveolar epithelial cells, and low expression levels of CRTAC1 are associated with decreased lung function [8]. It is considered a potential marker of multifactorial pulmonary fibrosis and may be useful for monitoring the extent of distal lung involvement in patients with COVID-19 [9]. Screening of the target gene CRTAC1 in relation to lung adenocarcinoma (LUAD) in the TCGA dataset showed that it is expressed at lower levels in tumors than in normal tissues, and high CRTAC1 expression is associated with a favorable prognosis in patients with LUAD, suggesting its role as a marker for the early diagnosis of LUAD [8]. These studies consistently show that CRTAC1 acts as a tumor suppressor in NSCLC; however, whether it is involved in the response to chemotherapy remains unknown. Exploring its role in chemotherapy sensitivity may be useful for the design of individualized treatment regimens for NSCLC patients.

The identification of molecular markers for predicting the efficacy of chemotherapy or the sensitivity to platinum drugs is important for designing effective tumor treatments, increasing chemotherapy sensitivity, and reducing drug toxicity, which are critical factors for prolonging patients’ survival. This study examined the effect of CRTAC1 on the efficacy of NSCLC chemotherapy and its mechanism of action to guide chemotherapy decisions and improve the survival benefit of patients in the future.

## Results

### CRTAC1 expression is correlated with chemosensitivity of cisplatin treatment in NSCLC

**Fig. 1:**
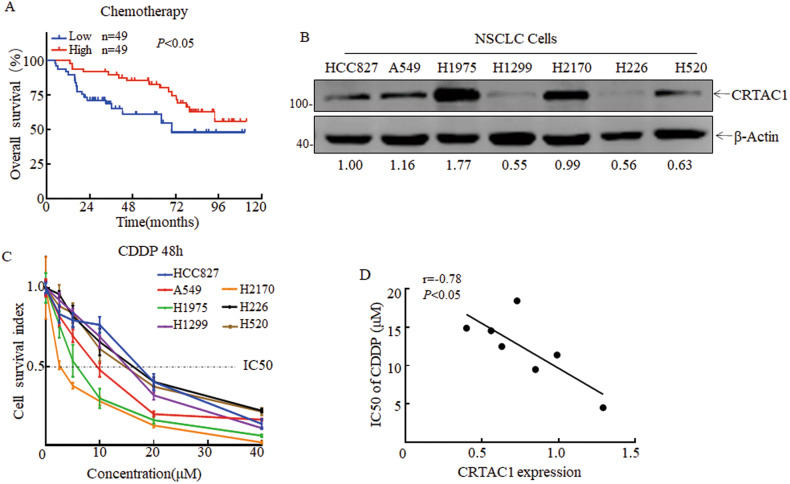
CRTAC1 is strongly associated with the response to chemotherapy in non-small cell lung cancer (NSCLC). **A** The overall survival of 98 patients with NSCLC treated with cisplatin-based chemotherapy in the GEO datasets was positively correlated with CRTAC1 expression levels. **B** The expression level of CRTAC1 protein in different NSCLC cell lines was detected by western blotting. The numbers below represent the ratio of the gray value of the CRTAC1 protein band relative to that of internal control. The H1299, H226, H520, and HCC827 cell lines were classified into the CRTAC1 low expression group, whereas A549, H2170, and H1975 cells were in the high expression group. **C** The viability of NSCLC cell lines treated with 0–40 µM cisplatin for 48 h was detected by the ATP assay, and the cisplatin IC50 values of different NSCLC cell lines were calculated. The IC50 for cisplatin was lower in the high CRTAC1 expression group than in the low expression group. **D** Pearson correlation analysis showed a significant negative correlation between the expression of CRTAC1 protein and the IC50 value of cisplatin in NSCLC cell lines. Low: low expression; High: high expression; *P* < 0.05 indicates a significant difference; r, correlation coefficient with (−) indicating a negative correlation.

Cisplatin-based chemotherapy is an effective treatment for patients with NSCLC. To analyze the relationship between the CRTAC1 expression and chemosensitivity in NSCLC, we used the gene chip probe ID 221204_s_at that represented by CRTAC1 to screen public microarray datasets for predicting the response of NSCLC patients receiving cisplatin-based chemotherapy from the Gene Expression Omnibus (GSE29013 and GSE14814). As shown in Fig. 1A, patients with high CRTAC1 expression had a significantly better response to chemotherapy than patients with low CRTAC1 expression (*P* < 0.05). The association of CRTAC1 expression with the response to chemotherapy was examined in NSCLC cell lines by calculating the 50% inhibitory concentration (IC50) values for cisplatin. The expression level of CRTAC in NSCLC cell lines was determined by Western blotting (Fig. 1B). Seven NSCLC cell lines were treated with different concentrations of cisplatin, and the IC50 values were calculated (Fig. 1C). The results showed that the IC50 for cisplatin was negatively correlated with CRTAC1 expression (Fig. 1D), indicating that CRTAC1 may be associated with chemosensitivity in NSCLC.

### CRTAC1 increases the sensitivity of NSCLC cells to cisplatin treatment in vitro

**Fig. 2:**
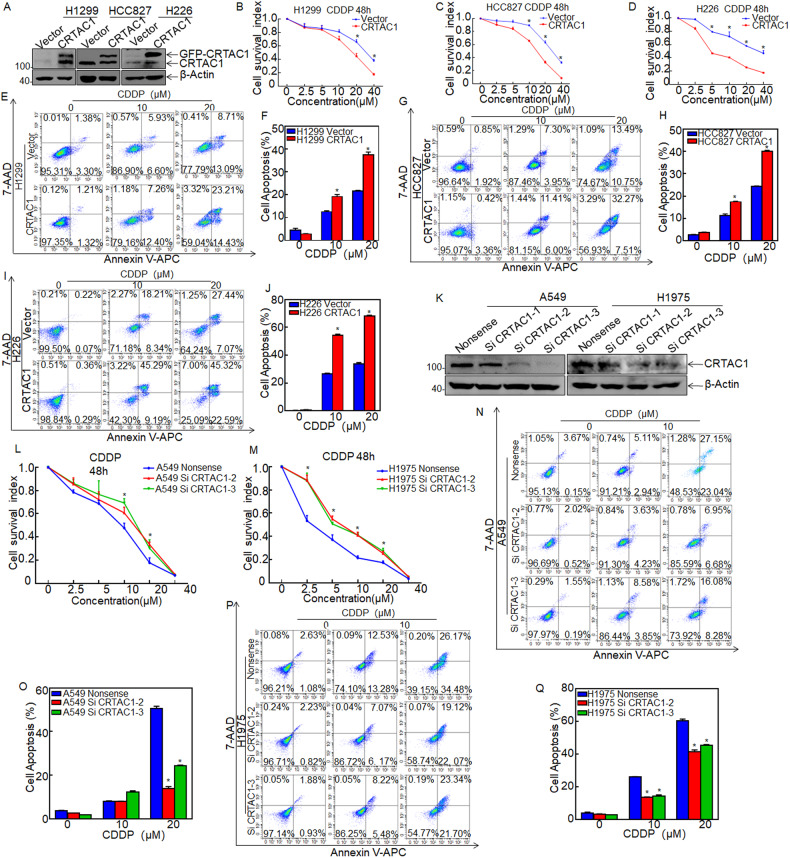
CRTAC1 increases the cisplatin sensitivity of NSCLC in vitro. **A** The stable CRTAC1-overexpressing NSCLC cell lines H1299, HCC827, and H226 (CRTAC1) were identified by western blotting. **B**–**D** The effect of CRTAC1 on the cell viability of cisplatin-treated NSCLC cells was analyzed using the ATP assay. **E**, **G**, **I** The effect of CRTAC1 overexpression on apoptosis of NSCLC cell lines after exposure to 0, 10, and 20 μM cisplatin for 48 h was detected by flow cytometry. **F**, **H**, **J** Statistical analysis of the rate of apoptosis of NSCLC cell lines overexpressing CRTAC1 after exposure to 0, 10, and 20 μM cisplatin for 48 h. Total apoptosis rate = ratio of early apoptotic cells (lower right) + ratio of late apoptotic cells (upper right). **K** Western blot analysis of the effect of transient CRTAC1 knockdown in the NSCLC cell lines A549 and H1975(siCRTAC1). **L**, **M** Effect of CRTAC1 knockdown on the viability of cisplatin-treated NSCLC cells was evaluated using the ATP assay. **N**, **P** Flow cytometry was used to detect the effect of CRTAC1 knockdown on cell apoptosis after exposure to 0, 10, and 20 μM cisplatin for 48 h. A representative image of cell apoptosis is shown. **O**, **Q** The apoptosis rates of NSCLC cell lines in the CRTAC1 knockdown group and the control group were calculated after exposure to 0, 10, and 20 μM cisplatin for 48 h. The results were plotted in a histogram. (*) indicates a significant difference (*P* < 0.05).

The role of CRTAC1 in chemotherapy sensitivity in NSCLC was examined using a panel of NSCLC cell lines with high cisplatin IC50 values. H1299, HCC827, and H226 (IC50 = 14.85 µM, 18.41 µM, and 14.52 µM, respectively) cell lines stably expressing CRTAC1 were generated, and control cells were transfected with empty vector. The expression of CRTAC1 was determined by Western blotting (Fig. 2A). ATP assays showed that chemosensitivity to cisplatin was significantly increased in CRTAC1-overexpressed cells in comparison to that observed in vector control cells (Fig. 2B–D). The effect of CRTAC1 on cell apoptosis was analyzed by flow cytometry, which showed a greater number of Annexin V positive cells, indicating apoptotic cells, in CRTAC1-overexpressing cells than in vector controls after treatment with CDDP (Fig. 2E, I, G). Consistently, CDDP treatment induced cellular apoptosis more significantly in CRTAC1-overexpressed cells than in vector transfectants (Fig. 2F, J, H).

To further explore the contribution of CRTAC1 expression to cisplatin sensitivity, different siRNAs were employed to knock down CRTAC1 in NSCLC cell lines with lower IC50 values for cisplatin (A549, IC50 = 9.495 µM and H1975, IC50 = 4.519 µM). Western blot analysis showed that siRNA2 and siRNA3 effectively silenced CRTAC1 expression (Fig. 2K). Assessment of cell viability using the ATP assay showed that the IC50 of cisplatin was higher in CRTAC1 knockdown cells than in scramble control cells (Fig. 2L, M). These data indicated that CRTAC1 increased the chemosensitivity of NSCLC cells to cisplatin. Cell apoptosis measured using the Annexin V/7-AAD assay also showed that cells transfected with siRNA against CRTAC1 had a significantly lower rate of apoptosis in response to cisplatin than that in scramble control cells, which was consistent with the results of the ATP assay (*P* < 0.05, Fig. 2N–Q).

### CRTAC1 overexpression increases cisplatin chemosensitivity in NSCLC in vivo

**Fig. 3:**
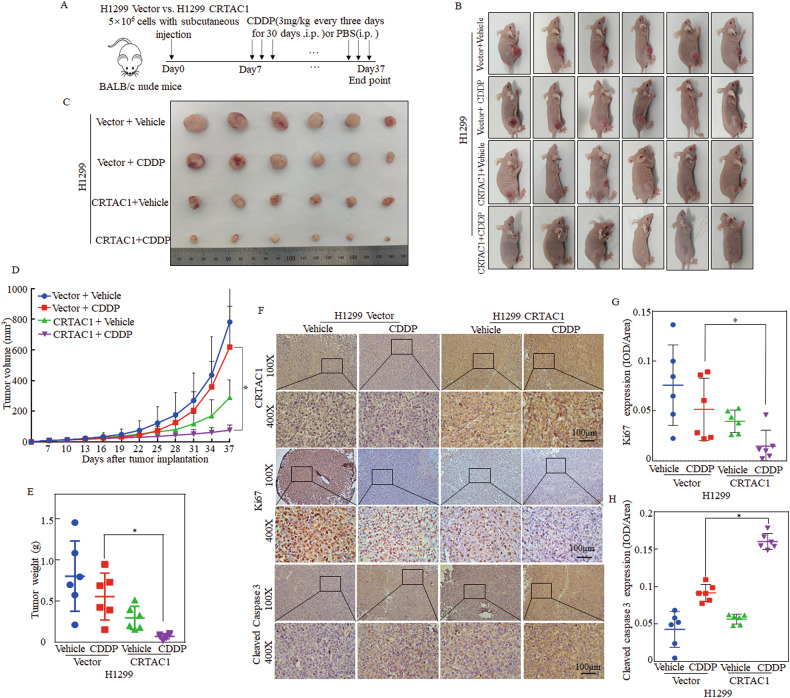
CRTAC1 increases the chemosensitivity of NSCLC to cisplatin in vivo. **A** H1299 (CRTAC1) and H1299 (Vector) cells were subcutaneously injected into the right back of nude mice at a density of 5 × 10^6^. Seven days after injection, when the subcutaneous tumor was palpable, the Vector and CRTAC1 groups were randomly divided into two subgroups, with six mice in each group. Patients in the CDDP group were intraperitoneally injected with cisplatin (3 mg/kg), whereas those in the vehicle group were intraperitoneally injected with PBS solution containing the same amount of DMF control solvent once every 3 days (×10). **B** After 30 days, four groups of tumor-bearing nude mice were photographed. **C** The nude mice were sacrificed, and the subcutaneous tumors were excised and photographed. **D** The size of the transplanted tumors was measured every 3 days. The length and width of the subcutaneous tumors were measured, and volume was calculated using the following formula: volume = 1/2 × width × width × length. The results were recorded in a dynamic monitoring map. **E** Chart of the weight of subcutaneous tumors. **F** Tumors were paraffin-embedded, cut into sections, and subjected to immunohistochemical staining. **G** Statistical chart of Ki67 immunohistochemical results in each group. **H** Statistical chart of cleaved caspase3 immunohistochemical results in each group. (*) indicates a significant difference (*P* < 0.05).

To explore the role of CRTAC1 in cisplatin sensitivity in vivo, a subcutaneous xenograft model was established by injecting CRTAC1-overexpressing cells and vector control cells into BALB/c-nude mice. The timeline and treatment of the xenograft mouse model were shown in Fig. 3A. Palpable tumors formed after 7 days, and mice were randomly divided into two subgroups, including the CRTAC1 overexpression and vector control groups (*n* = 6). The experimental group was injected intraperitoneally with cisplatin at a dose of 3 mg/kg, whereas the solvent control group received the same amount of PBS once every 3 days for 30 days.

Cisplatin inhibited the tumorigenic ability of NSCLC cells more effectively in the CRTAC1 overexpression group than in the vector control group (Fig. 3B, C). After cisplatin treatment, the mean NSCLC tumor size and weight were significantly lower in the CRTAC1 overexpression group than in the vector control group (Fig. 3D, E). Immunohistochemical staining showed that cisplatin downregulated Ki67 and upregulated cleaved caspase 3 markedly in CRTAC1-overexpressing tumors than in control tumors, indicating that overexpression of CRTAC1 dramatically attenuated cell proliferation and significantly promoted cell apoptosis (Fig. 3F–H). Taken together, these results indicated that CRTAC1 increases cisplatin sensitivity to cisplatin treatment in vivo.

### CRTAC1 promotes the sensitivity of NSCLC to cisplatin by inhibiting Akt1 expression

**Fig. 4:**
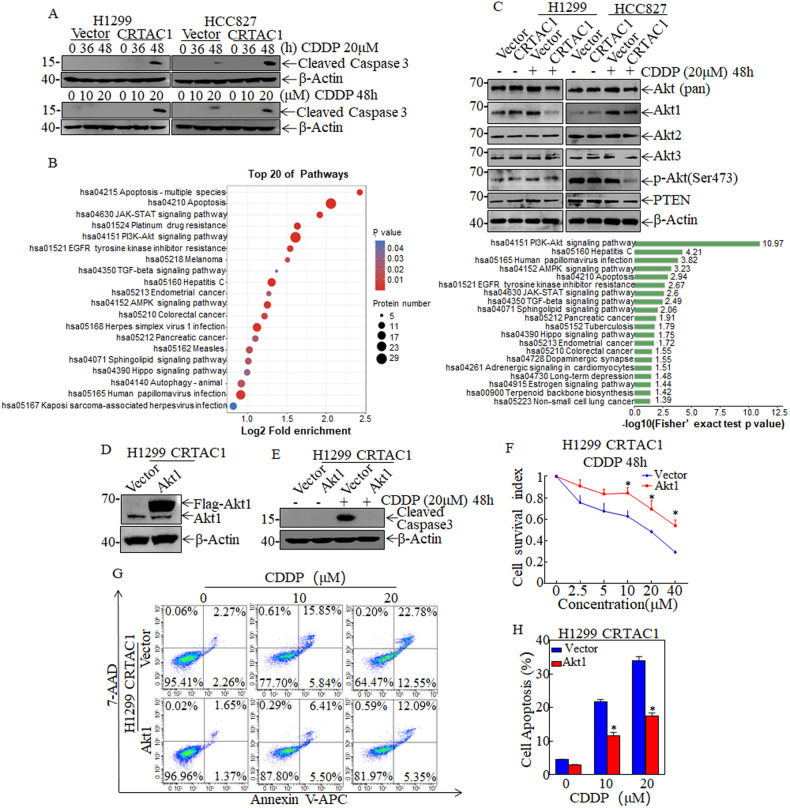
CRTAC1 increases the sensitivity of NSCLC to cisplatin by inhibiting the PI3K/Akt pathway. **A** Cleaved caspase3 was detected by western blotting in NSCLC (CRTAC1) cells and NSCLC (Vector) cells treated with 20 μM cisplatin for different times (0, 36, and 48 h) or different concentrations (0–40 μM) of cisplatin for 48 h. **B** KEGG pathway enrichment analysis of differential protein expression in H1299 (CRTAC1) and H1299 (Vector) cells treated with cisplatin. **C** H1299/HCC827 (CRTAC1) cells were subjected to western blotting to detect the effect of cisplatin on the expression of Akt (pan), Akt1, Akt2, Akt3, p-Akt1(Ser473) and PTEN. **D** Akt1 overexpression efficiency was confirmed in H1299 (CRTAC1) cells by western blotting. **E** The change of apoptosis-related proteins in H1299 (CRTAC1/Akt1) cells after cisplatin treatment was determined by western blotting. **F** H1299 (CRTAC1/Akt1) cells were treated with cisplatin to detect changes in cellular viability using the ATP assay. **G** The apoptosis rate of H1299 (CRTAC1/Akt1) cells treated with cisplatin was detected by flow cytometry. **H** The apoptosis rate of H1299 (CRTAC1/Akt1) cells treated with cisplatin was expressed as a statistical chart. (*) indicates a significant difference (*P* < 0.05).

Cysteine proteases play an essential role in cytotoxic drug-induced apoptosis in NSCLC. The pro-apoptotic protein caspase 3 acts as a dominant execution protease in tumor cell apoptosis, and the therapeutic response rate of NSCLC cells is correlated with the expression level of cleaved caspase 3. We showed that the expression level of cleaved caspase 3 was higher in CRTAC1-overexpressing cells than in vector control cells in response to cisplatin treatment at 20 μM for 48 h, as determined by Western blotting. These data suggested that CRTAC1 sensitized NSCLC cells to cisplatin at the indicated concentration and treatment time (Fig. 4A). To examine the molecular mechanism underlying the effect of CRTAC1 on increasing cisplatin sensitivity, protein expression profiles were compared between CRTAC1-overexpressing cells and vector control cells using iTRAQ quantitative proteomics after cisplatin treatment. We identified 811 differentially expressed proteins between the two groups. The biological functions of these proteins were examined by KEGG pathway enrichment analysis, which showed that multiple signaling pathways were enriched, including the Akt signaling pathway. Fisher’s exact test *p*-values for this pathway suggested that there was a significant correlation between Akt signaling and CRTAC1-mediated cisplatin sensitivity in NSCLC cells (Fig. 4B). The Akt signaling pathway regulated downstream cell survival and apoptosis pathways, which was consistent with the finding that CRTAC1 enhanced cisplatin-induced apoptosis. Therefore, we detected the expression of regulators of the PI3K-Akt signaling pathway. The western blotting results in Fig. 4C showed that the ectopic expression of CRTAC1 inhibited Akt1 expression in H1299 and HCC827 cells upon cisplatin treatment without affecting Akt3 and PTEN expression significantly but only inhibited Akt2 expression in HCC827 cells. The decrease in Akt expression further led to a decrease in Akt phosphorylation. These results indicated that CRTAC1 and cisplatin acted synergistically to decrease the activity of the Akt signaling pathway in NSCLC cells.

To confirm that Akt1 is involved in the CRTAC1-mediated regulation of chemosensitivity in NSCLC, Akt1 was ectopically expressed in CRTAC1-overexpressing cells (Fig. 4D). Akt1-overexpressing cells were treated with cisplatin, and the level of cleaved caspase 3 was determined. Akt1 overexpression significantly downregulated cleaved caspase 3 in H1299 cells despite the presence of CRTAC1 (Fig. 4E). Next, we treated Akt1-overexpressing cells with different concentrations of cisplatin for 48 h and measured cell viability and cell apoptosis. Akt1 overexpression suppressed the effect of CRTAC1 to increase the viability of H1299 cells in the presence of cisplatin and decreased cisplatin-induced apoptosis (Fig. 4F–H). In addition, we investigated the effect of Akt inhibitor on chemotherapy sensitivity of NSCLC cells, and the results showed that the addition of the Akt inhibitor A-44365 enhanced chemotherapy sensitivity and cisplatin-induced apoptosis in H1299 and HCC827 cells (Supplementary Fig. S1). Collectively, these data suggested that Akt1 was an important downstream effector of CRTAC1-mediated regulation of chemotherapy sensitivity in NSCLC.

### STUB1 attenuates CRTAC1-mediated chemosensitivity of NSCLC cells to cisplatin treatment by promoting the ubiquitination and degradation of Akt1

**Fig. 5:**
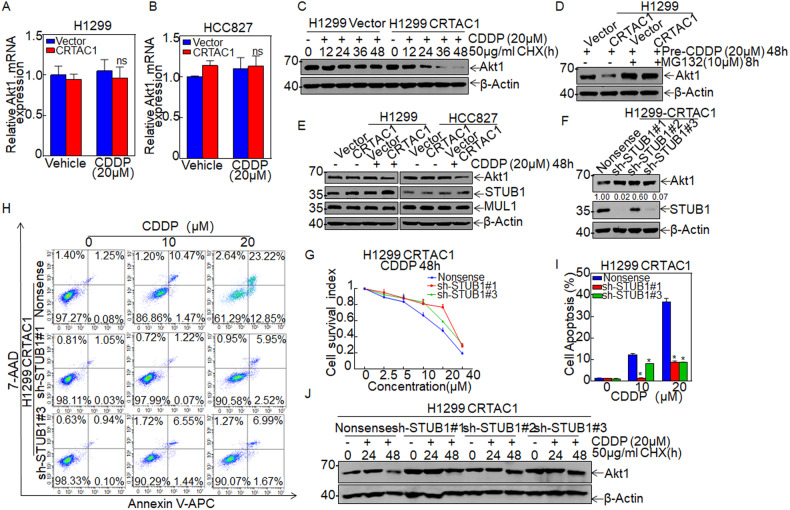
STUB1 attenuates the CRTAC1-mediated chemosensitivity of NSCLC cells by promoting the ubiquitination and degradation of Akt1 after cisplatin treatment. **A**, **B** The mRNA level of Akt1 in H1299/HCC827 (CRTAC1/vector) cells treated with cisplatin was detected by qRT-PCR. **C** The degradation rate of Akt1 was examined in H1299 (CRTAC1/vector) cells by western blotting after treatment with 50 μg/ml Cycloheximide and incubation with 20 μM cisplatin for 48 h. **D** The accumulation of Akt1 in H1299 (CRTAC1/vector) cells was examined by western blotting after treatment with 10 μM MG132 and 20 μM cisplatin. **E** The expression of the E3 ligases STUB1 and MUL1, which regulate the degradation of Akt1, was detected by western blotting in H1299 (CRTAC1/vector) cells. **F** Akt1 and STUB1 expression in H1299 (CRTAC1/shSTUB1#1, #2, #3) cells were detected by western blotting. **G** The killing effect of cisplatin in H1299 (CRTAC1/shSTUB1#1, #3) cells was examined using the ATP assay. **H**, **I** Cisplatin-induced apoptosis in H1299 (CRTAC1/shSTUB1#1, #3) cells was detected by flow cytometry. **J** The degradation rate of Akt1 was monitored by western blotting in H1299 (CRTAC1/shSTUB1#1, #2, #3) cells co-treated with CHX and cisplatin. Data were expressed as the mean ± SD, and an asterisk (*) indicates a significant difference (*P* < 0.05).

To clarify the regulatory relationship between CRTAC1 and Akt1, we first determined whether CRTAC1 affects Akt1 expression at the mRNA level by real-time PCR. The results showed that it was no significant difference in Akt1 mRNA expressions in response to cisplatin between the CRTAC1 overexpression group and the vector control group (Fig. 5A, B). Next, we inhibited protein synthesis using Cycloheximide (CHX) to determine whether CRTAC1 affects Akt1 degradation upon cisplatin treatment. As shown in Fig. 5C, CRTAC1 accelerated Akt1 degradation upon cisplatin treatment. The proteasome inhibitor MG132 was used to examine whether CRTAC1 regulates the abundance of Akt1 upon cisplatin treatment. The results showed that Akt1 accumulated to the same level in CRTAC1-overexpressing and vector control cells (Fig. 5D). These data suggested that CRTAC1-induced Akt1 degradation was mediated by the proteasome pathway.

The ubiquitin E3 ligases STIP1 homology and U-Box containing protein 1 (STUB1 /CHIP) and Mitochondrial E3 ubiquitin protein ligase (MUL1) catalyze the degradation of Akt1 in a proteasome-dependent manner. As shown in Fig. 5E, MUL1 protein expression did not change markedly in response to cisplatin treatment or in CRTAC1 overexpression cells without CDDP treatment, whereas the expression of STUB1 was slightly elevated in the CRTAC1/CDDP group in comparison to the other control groups. To determine whether STUB1 is involved in the effect of CRTAC1 on the chemosensitivity of NSCLC, three shRNA plasmids targeting different STUB1 sites were stably transfected into H1299 cells with overexpressed CRTAC1 to knock down STUB1 and then knockdown of STUB1 restored Akt1 protein expression (Fig. 5F). The ATP assay (Fig. 5G) and the apoptosis assay (Fig. 5H, I) showed that knockdown of STUB1 inhibited the effect of cisplatin on apoptosis, thereby decreasing the chemosensitivity of cells to cisplatin treatment in H1299-CRTAC cells. Knockdown of STUB1 also decreased the degradation rate of Akt1 in response to cisplatin treatment compared with the scramble control group (Fig. 5J). These results demonstrated that STUB1 acted as an intermediate effector molecule in the regulation of Akt1 degradation in CRTAC1-overexpressed cells upon cisplatin treatment, thereby playing an important role in the CRTAC1-mediated regulation of the chemosensitivity of NSCLC to cisplatin treatment.

### CRTAC1 promotes STUB1 transcription by enhancing NFATC1 activation to increase STUB1 promoter activity upon cisplatin treatment

**Fig. 6:**
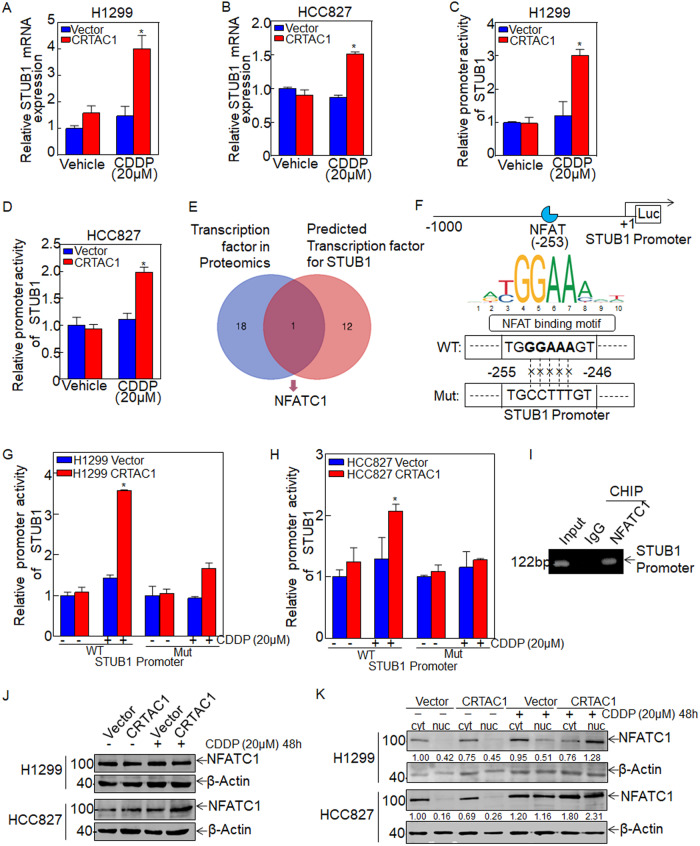
CRTAC1 promotes STUB1 transcription by regulating NFAT to enhance STUB1 promoter activity upon cisplatin treatment. **A**, **B** STUB1 mRNA in H1299/HCC827 (CRTAC1/vector) cells treated with cisplatin was detected by qRT-PCR. **C**, **D** A dual-luciferase reporter assay was used to detect the promoter activity of STUB1 in H1299/HCC827 (CRTAC1/vector) cells treated with cisplatin. **E** Differentially expressed transcription factors screened from the proteomics results were intersected with the transcription factors in the STUB1 promoter region predicted from the PROMO database. **F** Schematic representation of specific binding sites and mutation sites for NFAT binding to the STUB1 promoter region. **G**, **H** A dual-luciferase reporter assay was used to detect the promoter activity of mutant and wild-type STUB1 in H1299/HCC827 (CRTAC1/vector) cells treated with cisplatin. **I** A ChIP assay was used to identify the binding between NFAT and STUB1 promoter in H1299 cells. **J** H1299/HCC827 (CRTAC1) cells were subjected to western blotting to detect the effect of cisplatin on the expression of NFATC1. **K** Nucleo-plasmic separation assay was performed in H1299/HCC827 (CRTAC1) cells to detect the effect of cisplatin on NFATC1 nuclear localization. The numbers below represent the ratio of the gray value of the NFATC1 protein band relative to that of internal control. (*) indicates a significant difference.

To elucidate the molecular mechanism underlying the effect of CRTAC1 on promoting STUB1 protein expression, the mRNA levels of STUB1 were assessed in CRTAC1-overexpressing cells and vector control cells with and without cisplatin treatment. As shown in Fig. 6A, B, cisplatin significantly upregulated STUB1 mRNA expression in CRTAC1-overexpressing cells. A luciferase reporter gene assay was performed to detect STUB1 promoter activity and to explore the regulatory relationship between CRTAC1 and STUB1 upon cisplatin treatment. The results showed that overexpression of CRTAC1 significantly increased the promoter activity of STUB1 upon cisplatin treatment (Fig. 6C, D). These results indicated that CRTAC1 upregulated STUB1 by increasing STUB1 promoter activity under the effect of cisplatin.

Proteomic analysis combined with the prediction results in PROMO database to identify transcription factors associated with CRTAC1 biological functions suggested that Nuclear factor of activated T cells 1 (NFATC1 or NFAT2) might be the only transcription factor involved in the regulation of STUB1 expression by CRTAC1 at the transcriptional level (Fig. 6E). The specific binding site of NFAT is shown in Fig. 6F. To confirm that NFAT is the main effector molecule regulating the promoter activity of the STUB1 gene, the binding site GGAAA of NFAT in the STUB1 promoter region was mutated to CCTTT and a dual-luciferase reporter gene assay was performed. The results showed that the activity of the mutant STUB1 promoter was significantly lower than that of the wild type (Fig. 6G, H), indicating that NFAT can directly increase STUB1 promoter activity in CRTAC1-overexpressed NSCLC cells under the action of cisplatin. ChIP-PCR experiment further demonstrated the direct interaction between NFATC1 and endogenous STUB1 promoter (Fig. 6I). These results confirmed that NFAT was involved in the regulation of STUB1 gene transcription in NSCLC cells mediated by CRTAC1 under the action of cisplatin.

Next, we examined the effect of CRTAC1 on NFATC1 protein expression with or without cisplatin treatment, and the results showed that CRTAC1 upregulated NFATC1 expression in cisplatin-treated HCC827 cells but slightly downregulated NFATC1 expression in cisplatin-treated H1299 cells (Fig. 6J), indicating that CRTAC1 may not promote STUB1 transcription by upregulating NFATC1 expression. Since activated NFATC1 exerts signal transduction function by translocating to the nucleus [10], we investigated NFATC1 nuclear localization in CRTAC1-overexpressing cells and vector control cells with or without cisplatin treatment. As shown in Fig. 6K, CRTAC1 significantly promoted the nuclear localization of NFATC1 upon cisplatin treatment, indicating that CRTAC1 promotes STUB1 transcription by enhancing NFATC1 activation upon cisplatin treatment.

### CRTAC1 increases cisplatin-induced intracellular calcium levels by releasing calcium through the RyR channel

**Fig. 7:**
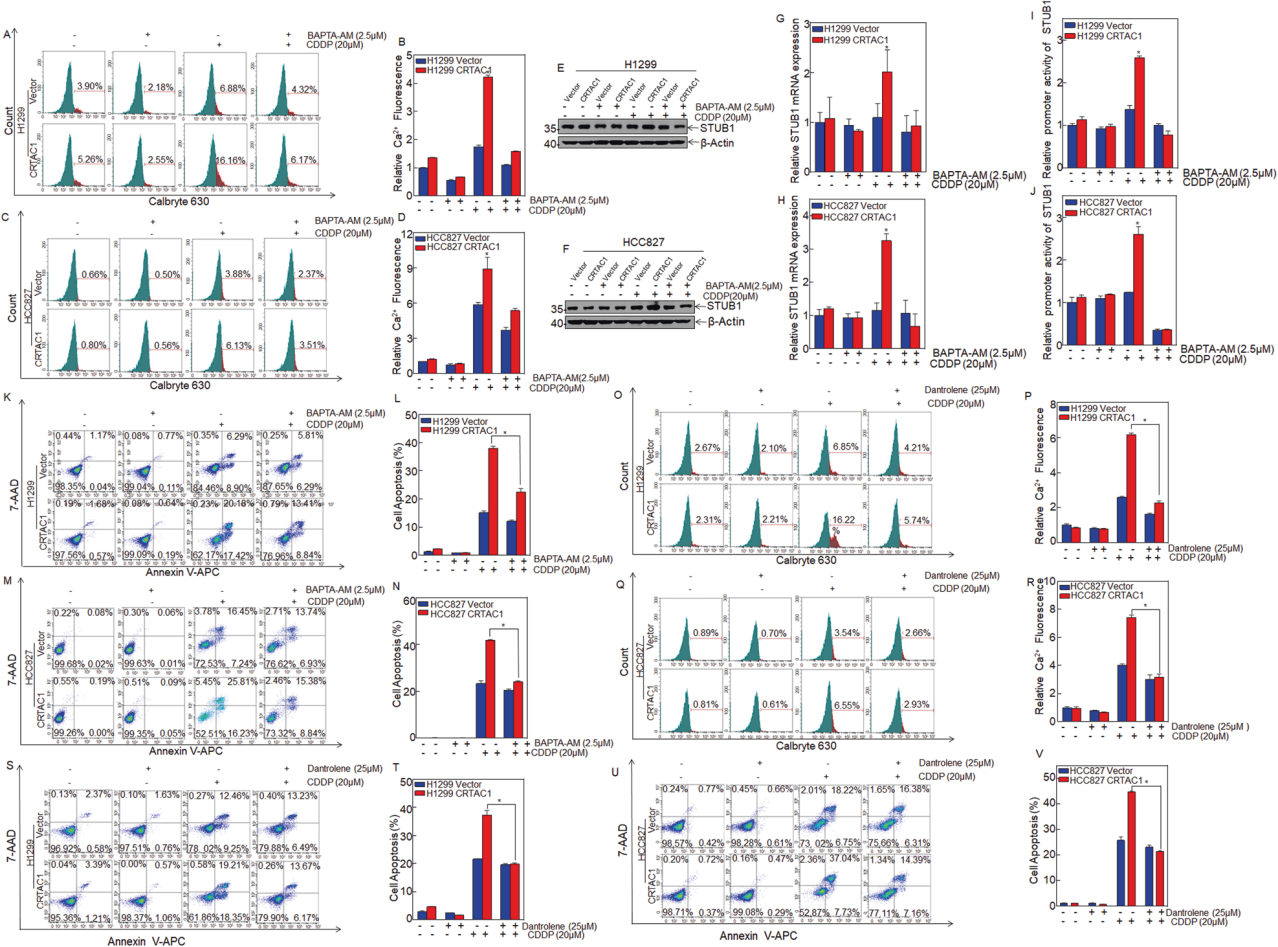
CRTAC1 increases cisplatin-induced intracellular calcium levels by releasing calcium through the RyR channel. **A**, **C** Calbryte 630 staining was used to detect the intracellular calcium level in H1299/HCC827 (CRTAC1/vector) cells pretreated with 2.5 μM BAPTA for 30 min and 20 μM CDDP for 48 h. **B**, **D** The relative calcium level was calculated in H1299/HCC827 (CRTAC1/vector) cells. **E**, **F** H1299/HCC827 (CRTAC1/vector) cells were pretreated with 2.5 μM BAPTA for 30 min and 20 μM CDDP for 48 h, and STUB1 expression was detected by western blotting. **G**, **H** H1299/HCC827 (CRTAC1/vector) cells were pretreated with BAPTA (2.5 μM) for 30 min and 20 μM CDDP for 48 h, and STUB1 mRNA expression was detected by qRT-PCR. **I**, **J** The activity of the STUB1 promoter was detected using a dual-luciferase reporter assay in cells treated with BAPTA and cisplatin. **K**, **M** H1299/HCC827 (CRTAC1/vector) cells were pretreated with BAPTA and treated with cisplatin, and apoptosis was detected by flow cytometry. **L**, **N** Statistical analysis of apoptosis in H1299/HCC827 (CRTAC1/vector) cells treated with BAPTA and cisplatin. **O**–**R** H1299/HCC827 (CRTAC1/vector) cells were pretreated with dantrolene (25 μM) for 2 h and incubated with cisplatin (20 μM) for 48 h. The relative calcium level was measured by flow cytometry. **S**–**V** H1299/HCC827 (CRTAC1/vector) cells were pretreated with dantrolene (25 μM) for 2 h and treated with cisplatin (20 μM) for 48 h, and apoptosis was detected by flow cytometry. (*) indicates a significant difference (*P* < 0.05).

The transcriptional activity of NFAT is regulated by the level of intracellular calcium, and calcium ions play an important role in signal transduction pathways that regulate gene transcription [11, 12]. Anticancer drugs such as cisplatin regulate intracellular Ca^2+^ levels and trigger calcium-dependent apoptosis [13]. CRTAC1, as a calcium-binding protein, could increase intracellular Ca^2+^ levels [14]. We hypothesized that the synergistic effect of CRTAC1 and cisplatin might be mediated by the co-regulation of calcium signals, thereby affecting the transcription of downstream genes and modulating the sensitivity of NSCLC cells to chemotherapy. In this study, the intracellular Ca^2+^ level was first detected by Calbryte 630 staining to evaluate its relationship with the expression of CRTAC1 in NSCLC cells. The intracellular Ca^2+^ level was higher in CRTAC1-overexpressing NSCLC cells than in the controls, and treatment with cisplatin significantly increased the intracellular Ca^2+^ level in the CRTAC1-overexpressing group (Fig. 7A–D). The intracellular Ca^2+^ level can directly increase the activity of the transcription factor NFAT. Treatment with the calcium chelator BAPTA to reduce the intracellular Ca^2+^ level downregulated STUB1 in CRTAC1-overexpressing NSCLC cells treated with cisplatin at the protein (Fig. 7E, F) and mRNA levels (Fig. 7G, H). STUB1 promoter activity was also blocked (Fig. 7I, J). These results suggested that CRTAC1 promoted the NFAT-mediated transcription of STUB1 by increasing cisplatin-induced intracellular calcium levels, thereby upregulating STUB1 expression. The results of the apoptosis assay showed that treatment with BAPTA significantly decreased the apoptosis rate of NSCLC cells in the CRTAC1 overexpression group treated with cisplatin (Fig. 7K–N). These results indicated that CRTAC1 may promote cisplatin-induced apoptosis by regulating intracellular Ca^2+^ levels in NSCLC cells.

The endoplasmic reticulum (ER) is an organelle that stores intracellular calcium ions, and its regulation of intracellular calcium concentration is mediated by the RyR and IP3R channels to release calcium ions from the ER lumen into the cytoplasm. To determine whether the increase in intracellular calcium in CRTAC1-overexpressing cells was mediated by the ER calcium channel, two different calcium channel blockers were selected, dantrolene and 2-aminoethoxydiphenyl borate (2-APB), which are inhibitors of RyR and IP3Rs, respectively. Treatment with dantrolene decreased intracellular Ca^2+^ levels in CRTAC1-overexpressing NSCLC cells, whereas 2-APB had no effect (Fig. 7O–R and Supplementary Fig. S1). Consistent with the intracellular calcium results, dantrolene treatment decreased the apoptosis rate of CRTAC1-overexpressing NSCLC cells, indicating that overexpression of CRTAC1 in NSCLC cells increases the intracellular calcium concentration induced by cisplatin through the RyR channel, thereby enhancing cisplatin-induced apoptosis.

## Discussion

Chemotherapy is widely used in the treatment of NSCLC, and the standard first-line chemotherapy regimen for NSCLC is cisplatin-based doublet chemotherapy. The drug combination and the dose and course of treatment are largely based on years of clinical experience and are determined by the type and malignancy of the cancer and the age and health of the patient. Because treatment is not targeted, establishing a reasonable treatment plan to achieve an optimal effect is difficult. A small number of patients treated with platinum-based chemotherapy achieve a complete response; many patients experience toxic side effects, and improper medication may even aggravate the disease. In the current era of precision medicine and considering advances in detection technology, designing an effective treatment strategy to predict the sensitivity of cisplatin before chemotherapy would enable the identification of patients who would benefit from a cisplatin-based chemotherapy regimen. It will be the direction of personalized treatment in the future.

In this study, Gene Expression Omnibus (GEO) dataset analysis showed, for the first time, that cisplatin treatment in NSCLC patients with high CRTAC1 expression levels is associated with better overall survival, suggesting that CRTAC1 might play a role in the efficacy of cisplatin chemotherapy in NSCLC. These findings suggest that CRTAC1 is a potential molecular marker for predicting the efficacy of chemotherapy in NSCLC. A significant negative correlation between the IC50 of cisplatin and the expression of CRTAC1 was detected in NSCLC cells treated with different concentrations of cisplatin for 48 h. CRTAC1 may play a role in predicting the chemosensitivity of NSCLC. The role of CRTAC1 in increasing the sensitivity of NSCLC cells to cisplatin was confirmed in vitro and in vivo by constructing cell and animal tumor-bearing models. The results showing that CRTAC1 increases the chemosensitivity of NSCLC are important for guiding the use of NSCLC chemotherapy strategies.

The CRTAC1 gene is located in human chromosome 10 long arm region 2 region 4 band 2 sub-band, and it shows tissue-specific expression. Transcriptomics and proteomics analyses show that CRTAC1 is expressed at high levels in normal lung tissues [15]. Low expression levels of CRTAC1 in specific tissues are related to the clinicopathological characteristics of the corresponding tissue and organ diseases [16–18]. CRTAC1 may serve as a marker for monitoring pulmonary fibrosis and new coronary pneumonia. CRTAC1 levels in the plasma and bronchoalveolar lavage fluid of such patients are reduced, and their lung function is decreased. Downregulation of CRTAC1 expression in lung cancer tissues predicts a poor prognosis, suggesting that it can act as a tumor suppressor [8]. The poor prognosis and low survival rate of tumor patients with low CRTAC1 expression may be related to the poor efficacy of various clinical treatments. These studies support the importance of CRTAC1 in modulating the efficacy of NSCLC chemotherapy.

In clinical chemotherapy treatment, cytotoxic injury-induced apoptosis, an important determinant of cisplatin sensitivity, is a process of programmed cell death that removes excess or damaged cells in an efficient, non-inflammatory manner. The cells are removed from the tissue, thus ensuring the stability of the tissue environment. In this study, we found that CRTAC1 promotes caspase-mediated apoptosis by increasing the cleavage of caspase3 in NSCLC cells exposed to cisplatin. However, solid tumors in different regions have different sensitivities to anticancer drugs, and the triggered signaling pathways are complex, resulting in decreased sensitivity of some cancer cells [19]. Abnormal activation or inhibition of intracellular signal transduction pathways is related to the resistance of tumor cells to chemotherapy [20]. However, tumor cells with high sensitivity to cisplatin can specifically inhibit survival signals and trigger cell death signals. The Akt signaling pathway is activated in many cancers and is involved in the regulation of biological processes related to tumor cell survival, differentiation, apoptosis, and metabolism [21]. Therefore, the molecular mechanism underlying decreased chemosensitivity caused by abnormal activation of the Akt pathway has received increased attention. Akt, a key downstream kinase of PI3K, is activated in a growth factor-dependent manner and can be altered under cellular stress conditions such as DNA damage or DNA-dependent protein kinases [22, 23].

Akt phosphorylated at the S473 site promotes activation of the PI3K/Akt signaling pathway [24]. The PI3K/Akt pathway can inhibit caspase-mediated apoptosis by upregulating phosphorylated Akt, ultimately improving tumor cell survival [25]. FK506-binding protein 51 acted as an intermediate bridge molecule to bind Akt and PHLPP and accelerated PHLPP dephosphorylation of p-Akt (S473), thereby increasing the sensitivity of pancreatic cancer cells to chemotherapy drugs [26, 27]. Downregulating Akt or inhibiting the activation of the PI3K/Akt signaling pathway through upstream regulators induces apoptosis and is a potentially beneficial adjuvant antitumor therapy [28–30]. Small-molecule inhibitors targeting this pathway, such as wortmannin and LY294002, which are common PI3K inhibitors, have been studied extensively. Wortmannin binds to the 110 kD catalytic subunit of PI3K, specifically inhibits PI3K, and inhibits the PI3K/Akt signaling pathway [31]. Wortmannin can also inhibit myosin light chain kinase (MLCK), which is not a specific effect. Wortmannin undergoes rapid decay or is degraded, and it is thus not suitable for clinical trials. LY294002 suppresses the enzymatic activity of PI3K through a competitive inhibition process and can selectively and indirectly inhibit the PI3K/Akt pathway in a reversible manner [32]. LY294002 also inhibits the activity of MLCK, PI3K, and phospholipase C, but not specifically [33]. Therefore, it is particularly important to identify molecules that specifically target Akt. This study elucidated a novel molecular mechanism by which CRTAC1 targets the major isoform of Akt, a key intermediate of the PI3K/Akt pathway. We showed that CRTAC1 inhibits Akt phosphorylation at S473 by downregulating Akt1 expression, providing information on the molecular regulatory network of Akt activation as well as new insight for the development of Akt inhibitors to improve the chemosensitivity of NSCLC.

Increasing evidence suggests that changes in Akt1 expression are caused by its post-translational modification. In this process, K48-linked ubiquitin chains bind to Akt1 and target it for 26S proteasomal degradation, which is the major step in the downregulation of Akt1 expression [34, 35]. Therefore, a comprehensive understanding of the role of post-translational modification in the regulation of Akt1 could help the design of new strategies for cancer therapy. STUB1 promotes the K48-linked ubiquitination and degradation of Akt1 to terminate its activation [36–38]. STUB1 is an E3 ubiquitin protein ligase and a cofactor that can interact with Hsp70, Hsp90, or other molecular chaperones to target misfolded chaperone substrates toward proteasomal degradation [39]. Since cisplatin was reported to cause the accumulation of unfolded or misfolded proteins [40], there might be a possibility that cisplatin causes misfolding of AKT1, which in turn leads to degradation by STUB1. In this study, we showed that CRTAC1 regulates the activity of STUB1 in promoting cisplatin-induced Akt1 ubiquitination and degradation. This study is the first to demonstrate the role of STUB1 in increasing cisplatin sensitivity in NSCLC cells, which improves our understanding of the function of STUB1.

This study demonstrated that CRTAC1 upregulated STUB1 mRNA expression by promoting STUB1 promoter activity under cisplatin treatment. According to the reported binding sites of transcription factors and the sequence of the STUB1 promoter region, comprehensive proteomic analysis showed that NFAT can directly bind to the promoter of STUB1 and induce STUB1 transcription. This is the first study showing that NFAT regulates the transcription and expression of STUB1, which provides a new understanding of the molecular mechanism by which NFAT regulates STUB1. NFAT is a well-established transcription factor regulated by calcium ions [11, 41]. Calcium ions are key signaling molecules in cells, and transcription factors are activated by calcium ions and undergo structural changes, thereby translocating to the nucleus to regulate gene transcription [42]. Increased intracellular calcium levels activate calcineurin and trigger the dephosphorylation of NFAT; this induces a conformational change in NFAT. NFAT exposes the nuclear localization sequence and the residues that will contact DNA, thereby translocating into the nucleus and increasing DNA binding activity to promote gene transcription [11, 43]. In this study, CRTAC1 increased the uptake of free calcium ions in NSCLC cells, and the combined CRTAC1 overexpression and cisplatin treatment induced a significant increase in the intracellular Ca^2+^ level. This increased the transcriptional activity of NFAT, thereby upregulating STUB1 protein expression. CRTAC1 regulates the uptake of intracellular free calcium ions in human lens epithelial cells, thereby promoting cell apoptosis [44], which is consistent with the results of this study. CRTAC1 has a C-terminal epidermal growth factor-like Ca^2+^ binding domain that mediates its involvement in calcium signal transduction pathways [45, 46]. These findings indicate that CRTAC1 can act as a regulator of intracellular calcium levels and plays an important role in improving the chemosensitivity of NSCLC.

The ER is a major store of intracellular calcium and releases free calcium under certain stress conditions [47]. In this study, overexpression of CRTAC1 in combination with cisplatin treatment increased intracellular calcium concentration. However, treatment with the RyR channel inhibitor dantrolene decreased the intracellular calcium concentration. Therefore, CRTAC1 and cisplatin jointly regulate the dynamic balance of calcium ions in the ER through RyR channels, thereby activating NFAT. However, the specific mechanism by which CRTAC1 cooperates with cisplatin to regulate intracellular calcium ions through RyR channels remains unclear, and further research is needed. At present, only the results indicated in the clinical patient dataset have been verified by in vitro and in vivo experiments. Clinical trials are needed to evaluate the use of CRTAC1 as a new biomarker for predicting the efficacy of cisplatin or as a new agent for the treatment of NSCLC.

In conclusion, this study demonstrated, for the first time, that CRTAC1 promotes cisplatin-dependent apoptosis in NSCLC and is a potential tumor molecular marker for predicting and improving cisplatin sensitivity in patients with NSCLC. CRTAC1 may thus improve the efficacy of cisplatin therapy. The development of small-molecule inhibitors or activators targeting NSCLC drug sensitivity based on the CRTAC1/RyR/Ca^2+^/NFAT/STUB1/Akt1 signaling pathway newly elucidated in this study will help guide the implementation of individualized treatment strategies for NSCLC patients.

## Materials and methods

### Cell lines and plasmids

BEAS-2B cells were cultured in Dulbecco’s modified Eagle’s medium (DMEM; #11995-065; Gibco, Grand Island, NY, USA). The NSCLC cell lines A549, H1299, H1975, HCC827, H2170, H226, and H520 were obtained from ATCC (Manassas, VA, USA). H1299, H1975, HCC827, H2170, H226, and H520 cells were cultured in RPMI 1640 (#R8768; Sigma-Aldrich, Milwaukee, WI, USA), and A549 cells were cultured in Ham’s F-12K (#21127-022; Gibco, Grand Island, NY, USA). Media were supplemented with 10% fetal bovine serum (FBS; #10437-028, Gibco). The CRTAC1 overexpression plasmid and the vector plasmid were purchased from Sunny Biotechnology (Shanghai, China). The different siRNAs targeting CRTAC1 and its scrambled control siRNA were purchased from Jinweizhi Biotechnology (Suzhou, China). The Akt1 overexpression plasmid and control plasmid were purchased from Miaoling Biotechnology (Wuhan, China). The STUB1 shRNA plasmid, the control plasmid, and the STUB1 promoter plasmid (full length, from −1020 to +1) were synthesized by Qingke Biotechnology (Beijing, China). The STUB1 promoter Mut plasmid was constructed by the author. Point mutation PCR was performed using the forward outside primers (F1) 5′-CTATCGATAGGTACCAGGGAGGCCCCGCCC CCA CT-3′, and (F2) 5′-TGAGGCATCTCACTGCCTTTGTCGAATGTGTGTGG-3′, and the reverse primers (R1) 5′-ATCGCAGATCTCGAGAGCTCCGCCGGA-3′, and (R2) 5′-CACACACATTCGACAAAGGCAGTGAGATGCCTCAC-3′. The PCR products were digested and cloned into pGL3-Basic (E1751; Promega, Madison, WI, USA) and verified by DNA sequencing.

### Cell transfection and generation of stable cell lines

Plasmids were introduced into cells using Polyjet^TM^ DNA in Vitro Transfection Reagent (SignaGen Laboratories). GFP-CRTAC1 stable expression cells and vector control cells were constructed by lentivirus infection and screened by puromycin. Lentivirus packaging and infection experiments were performed as described previously [48]. RiboFECT™ CP diluted in OptiMEM (Life Technology) was used to deliver the siRNA #1, 5′-GCAGUGCCUCGGAUAUCUUTT-3′, #2, 5′-GCCAAUU ACGCCUACGGUATT-3′, #3, 5′-UGGACCCAACCUGGUUCUGAATT-3′ targeting CRTAC1 into the cells according to the manufacturer’s instructions. The shRNAs targeting STUB1 were cloned into pLKO.1 and transfected into cells, then select stable transfection cells with G418 (Goldbio) for at least 3 weeks. The following three target sequences of STUB1 were used: shRNA-1, 5′-CCGGGAAGAGGAAGAAGCGAGACATCTCGAGATGTCTCGCTTCTTCCTCTT CTTTTTG-3′, shRNA-2, 5′-CCGGGCAGTCTGTGAAGGCGCACTTCTCGAGT AACTTTGAAAGAGGGAGCTTTTTTG 3′ shRNA-3, 5′-CCGGCGCGAAGAAG AAGCGCTGGAACTCGAGTTCCAGCGCTTCTTCTTCGCGTTTTTG 3′.

### Reagents and antibodies

Cisplatin (15663-27-1) was purchased from Sigma-Aldrich. Cycloheximide and MG132 were from Santa Cruz Biotechnology (Dallas, TX, USA). BAPTA-AM (S7534) was from Selleck Chemicals (Houston, TX, USA). Dantrolene (S80373) was obtained from Yuanye Bio-technology Co., Ltd (Shanghai, China). Antibody against CRTAC1 (Abcam Cat# ab102548, RRID: AB_10710442) was purchased from Abcam (Cambridge, UK). Antibodies against cleaved caspase3 (9661S), Akt (pan) (4685S), Akt1 (75692S), p-Akt (ser473) (4060S), and STUB1 (2080S) were obtained from Cell Signaling Technology (Boston, MA, USA). Antibodies against MUL1 (Proteintech Cat# 16133-1-AP, RRID: AB_2147111), NFATC1 (66963-1-Ig) and β-actin (HRP-60008) were from Proteintech (Wuhan, China).

### Bioinformatics analysis

The gene expression data and Kaplan–Meier survival plots for NSCLC patients were obtained from the GEO (Gene Expression Omnibus (GEO), RRID:SCR_005012) database (GSE29013 and GSE14814). The 4D label-free quantitative proteomics technology (PTM-Biolab, Hangzhou, China) was used to analyze the mass spectra of the samples and construct the specific protein database. The identified proteins were annotated in the KEGG (KEGG, RRID:SCR_012773) database and enriched by Fisher’s exact test.

### Western blot analysis

Cells were lysed in lysis buffer, and proteins were extracted. Proteins were separated by SDS-PAGE and transferred to PVDF membranes. The membranes were probed with the indicated primary antibodies, followed by incubation with AP-conjugated secondary antibodies. An enhanced ECF chemifluorescence system was used to detect the signals, and images were acquired on a phosphorimager (Typhoon FLA 7000; GE Healthcare, MA, USA).

### Cell viability assay

Cell viability was examined with the CellTiter-Glo^®^ luminescent cell viability assay kit (G7572, Promega). Cells (3 × 10^3^ cells/well) were seeded in 96-well plates with 200 μl of RPMI 1640 medium containing 10% FBS. Then, the cells were exposed to cisplatin at different concentrations for 48 h. Next, 25 μl CellTiter-Glo® reagent and 25 μl PBS were added per well on a vortex for 2 min. After incubating for 10 min to stabilize the luminescence signal, the plates were analyzed using a microplate luminous detector (LB960, Berthold, Germany).

### Apoptosis assay

Cell apoptosis was determined using a cellular apoptosis assay kit (AP105, Lianke Bio). Cells were treated with cisplatin for 48 h and resuspended in a binding buffer. Then, 5 μl Annexin V-APC and 10 μl 7-AAD were added sequentially and incubated for 5 min in the dark. The apoptosis rate was detected by flow cytometry (BECKMAN, USA).

### Nude mouse xenograft model

Animal experiments were performed at the animal institute of Wenzhou Medical University according to protocols approved by the Laboratory Animal Center of Wenzhou Medical University and the Laboratory Animal Ethics Committee of Wenzhou Medical University. Twenty-four female BALB/c athymic nude mice (3–4 weeks old) were obtained from GemPharmatech (GemPharmatech, RRID:SCR_017239; license number: SCXK 2018-0008; Nanjing, Jiangsu, China). After 1 week of growth, the nude mice were randomly divided into two groups, with 12 mice in each group. A total of 5 × 10^6^ H1299 Vector cells or H1299 CRTAC1 cells/100 µl PBS were injected subcutaneously into nude mice. When the subcutaneous tumors were palpable on day 7 after injection, the mice were randomly divided into two subgroups (*n* = 6), a CDDP group and a vehicle control group. The CDDP group was treated intraperitoneally with 3 mg/kg cisplatin every 3 days, and the vehicle group was treated intraperitoneally with PBS. After 30 days of treatment, the subcutaneous tumor tissues of nude mice were obtained.

### Immunohistochemistry (IHC)

Tissue samples were embedded in paraffin after gradient dehydration, and 4 μM slices were made. After xylene dewaxing and alcohol gradient rehydration (100%, 95%, 90%, 80%, 70%, and 50%), microwave antigen retrieval was performed in citrate buffer. After cooling to room temperature, the samples were treated with 3% H_2_O_2_ for 10 min and incubated for 30 min with 3% fetal bovine serum at room temperature. The tissue samples were then incubated overnight at 4 °C with antibodies against CRTAC1 (GTX119558, GeneTex), MKI67 (Ab16667, Abcam), and cleaved caspase3 (9661S, Cell Signaling Technology). Staining was performed with the Ready-to-Use SABC-POD Kit (SA1022; BOSTER Biological Technology, Wuhan, China). Immunostained images were acquired on a Nikon Eclipse Ni microsystem (DS-Ri2).

### qRT-PCR assay

Total RNA was isolated from cultured cells using the TRIzol reagent (Invitrogen, Carlsbad, CA, USA). RNA was reverse-transcribed into total cDNA using the PrimeScript^TM^ RT kit (#RR036A; TaKaRa, Kyoto, Japan) and then subjected to RT-PCR. mRNA expression levels were determined by qPCR on a Q6 real-time PCR System (Applied Biosystems, Carlsbad, CA, USA) with SYBR qPCR Master Mix (4309155, Applied Biosystems) using GAPDH as an internal loading control. The primers used in this study were as follows: human *Akt1* (forward: 5′-CTTGCTTTCAGGGCTGCTCA-3′ and reverse: 5′-TACACGTGCTGCCACAGGA TAC-3′); human *STUB1* (forward: 5′-CTCTCACGCTCCGCGGCAAT-3′ and reverse: 5′-GCCAAGGAGCAGCGGCTGAA-3′); and human *GAPDH* (forward: 5′-GACTC ATGACCACAGTCCATGC-3′ and reverse: 5′-CAGGTCAGGTCCACCACTGA-3′).

### Luciferase reporter assay

Luciferase reporters driven by the *STUB1* promoter (P-STUB1-WT and P-STUB1-Mut) and their control plasmid were co-transfected with pRL-TK (RRID: Addgene_11313) into cells. The relative luciferase activity of specific samples was evaluated using the Dual-Luciferase Reporter Assay System (E1500, Promega) and measured using a microplate luminous detector.

### ChIP assay

The SimpleChIP Enzymatic Chromatin IP Kit (9003, Cell Signaling Technology) was used for the ChIP assay. Briefly, 4 × 10^6^ H1299 cells for each sample were prepared. Normal rabbit IgG and NFATC1 antibodies were used in the control and experimental groups, respectively. Finally, the levels of the STUB1 promoter in the input and IP samples were detected by PCR, with primers as STUB1-122bp-Forword: 5′-AGGTGGTCGGGACAGGCTGTT -3′ and STUB1-122bp-Reverse: 5′-CTGCTGA GGTCTCTTCGGAA-3′.

### Nucleo-plasmic separation assay

The Thermo Fisher PARIS™ Kit was used to perform nucleo-plasmic separation experiments. Briefly, 5 × 10^5^ vector control cells and CRTAC1-overexpressed cells were seeded into a 10 cm dish. Upon adhesion, cells were starved with RPMI 1640 medium containing 0.1% FBS for 12 h, and then the medium was switched to the complete medium with or without 20 μM cisplatin for 48 h. After being treated with trypsinization, cells were collected, resuspended using 500 μl of ice-cold cell fractionation buffer, and then incubated on ice for 5–10 min. Upon incubation, samples were centrifuged at 4 °C and 500×*g* for 5 min, and the supernatant of them was collected as the cytoplasmic component. The pellet was then resuspended with 500 μl of ice-cold cell disruption buffer and incubated on ice for 5–10 min to obtain nuclei components.

### Intracellular Ca^2+^ concentration [Ca^2+^] measurement

Cells were trypsinized into single cell suspensions and incubated with 500 μl HBSS with 5 μM Calbryte™ 630 AM (AAT Bioquest, Inc., Sunnyvale, CA, USA) for 1 h at room temperature, according to the manufacturer’s instructions. Then, cells were washed twice with HBSS to remove excess dye and analyzed using a CytoFLEX flow cytometer to measure fluorescence intensity.

### Statistical analysis

Statistical analyses were performed using GraphPad Prism (GraphPad Prism, RRID:SCR_002798, version 7.0, Inc., La Jolla, CA, USA) software, and data were presented as the mean ± standard deviation. Differences between groups were analyzed by independent *t*-test, and *P* < 0.05 represented a statistically significant difference and indicated by *. Pearson correlation analysis was used to analyze the correlations between two groups, and the results were expressed by R. The value range of R was between −1 and +1. The R value of a negative correlation was −1 to 0, and 0 to +1 represented a positive correlation. Significant differences between the two groups were expressed by *P* < 0.05.

## Supplementary information

Supplymentary Figure S1

Supplemental Material_Original Western blots

aj-checklist

## Data Availability

The data that support the findings of this study are openly available in ProteomeXchange Dataset at (https://www.ebi.ac.uk/pride/archive/). Proteomics data accession number is PXD041782.
