# Supplementary material for: CRTAC1 enhances the chemosensitivity of non-small cell lung cancer to cisplatin by eliciting RyR-mediated calcium release and inhibiting Akt1 expression
Source: Cell Death Dis. 2023 Aug 26;14(8):563. doi: 10.1038/s41419-023-06088-1 (PMC10460435; doi:10.1038/s41419-023-06088-1)

Figure1B

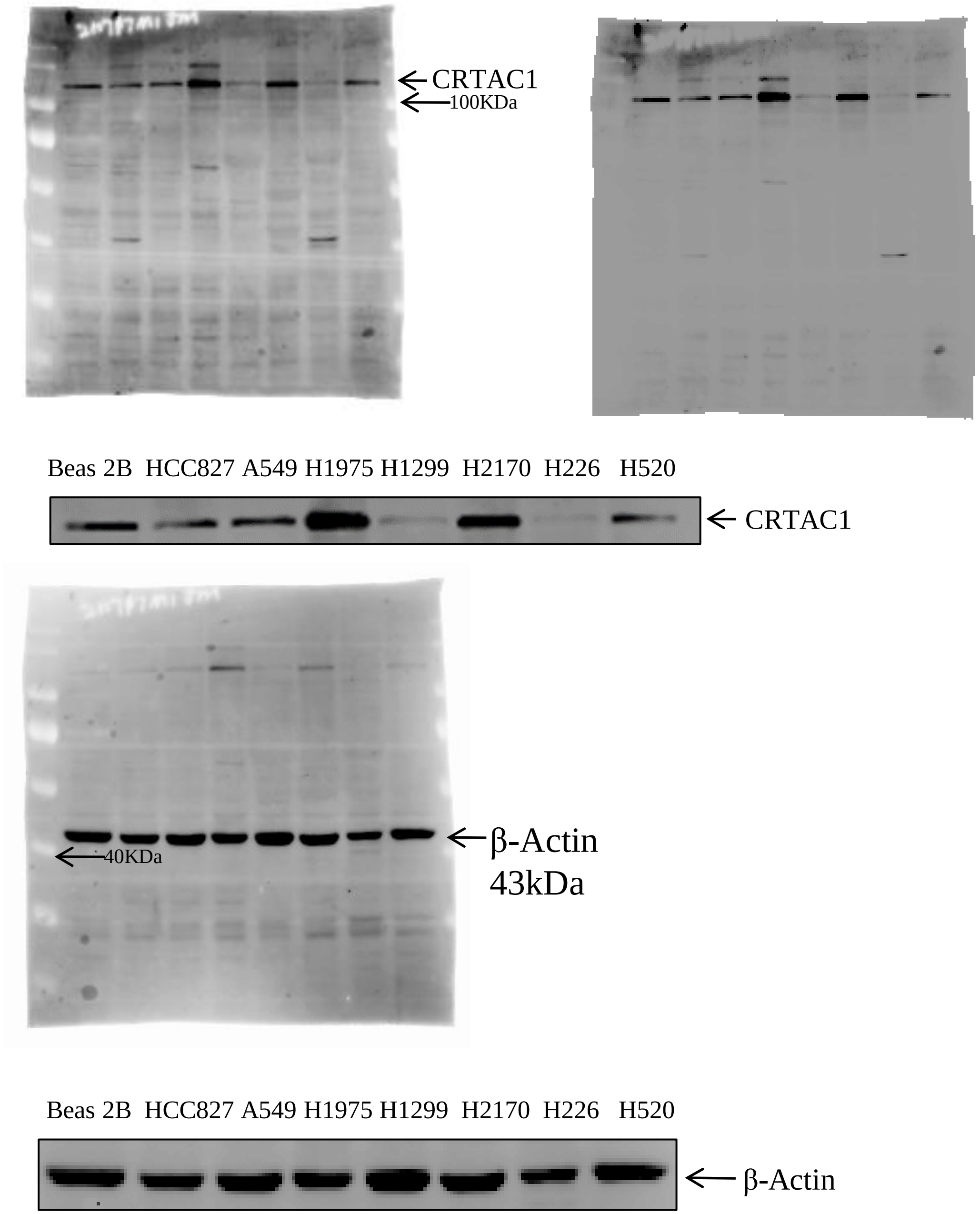

CRTAC1
100KDa
Beas 2B HCC827 A549 H1975 H1299 H2170 H226 H520
CRTAC1
β-Actin
43kDa
40KDa
Beas 2B HCC827 A549 H1975 H1299 H2170 H226 H520
β-Actin

B

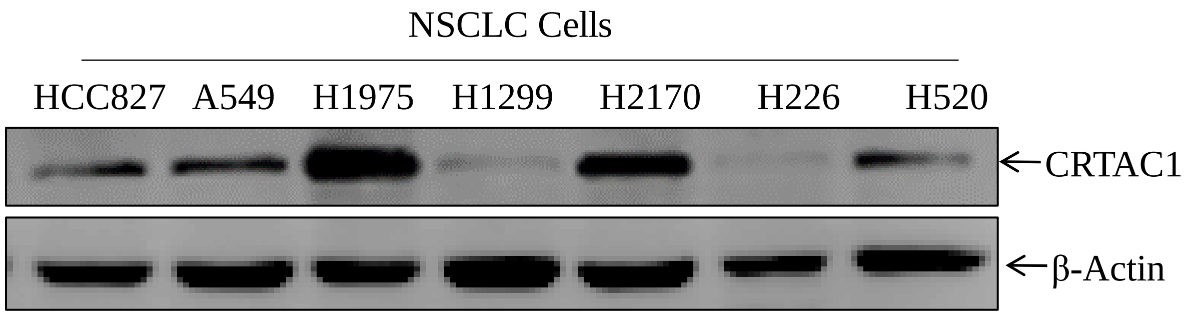

NSCLC Cells
HCC827 A549 H1975 H1299 H2170 H226 H520
CRTAC1
β-Actin

Figure2A

H1299-CRTAC1

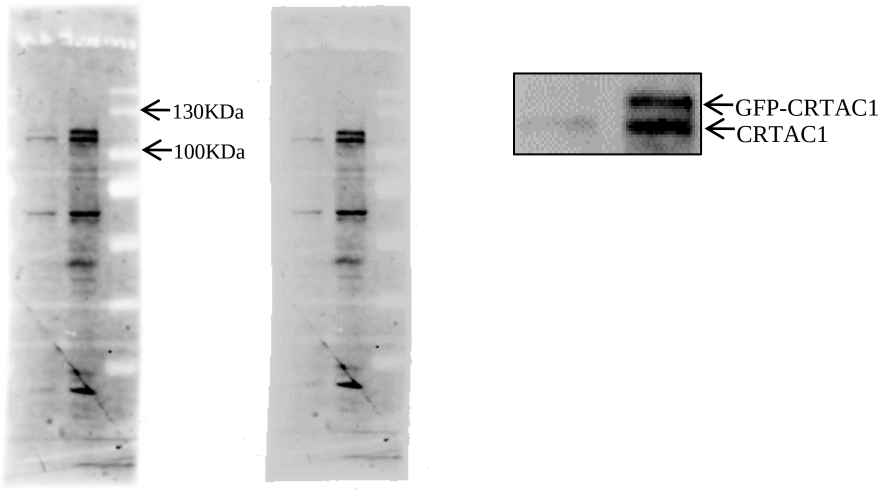

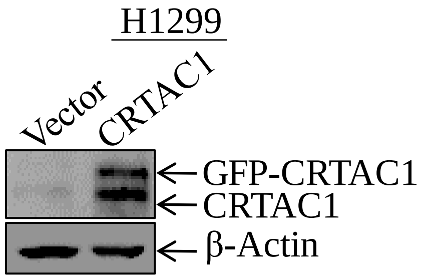

H1299-actin

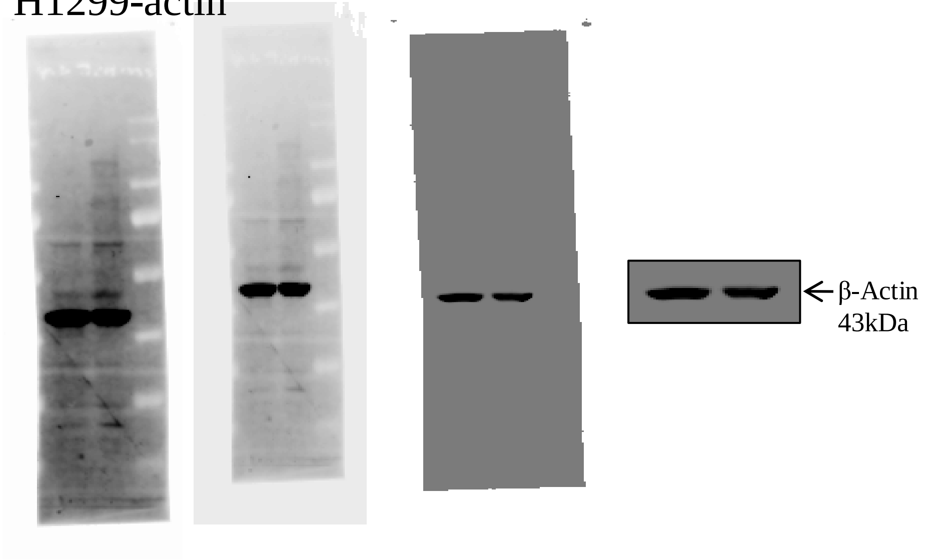

Figure2A

HCC827-CRTAC1

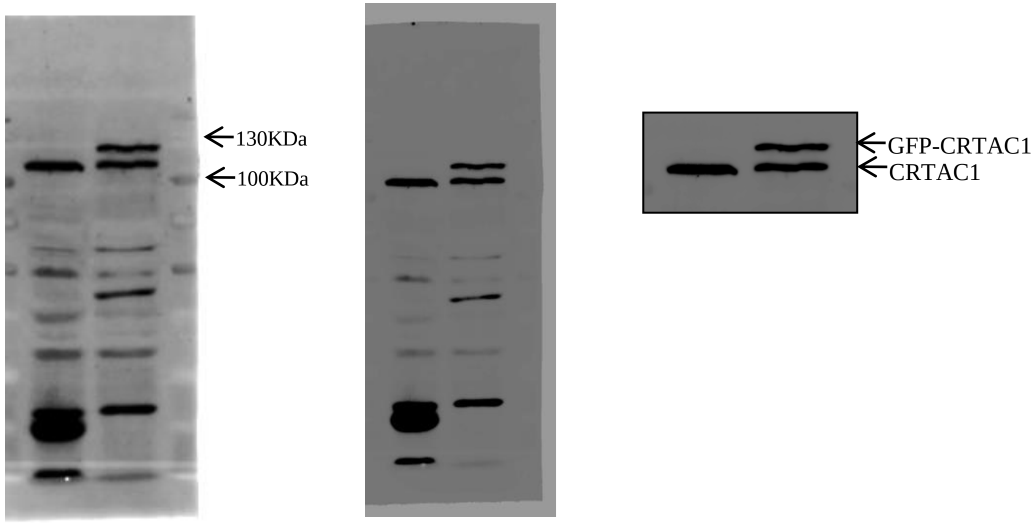

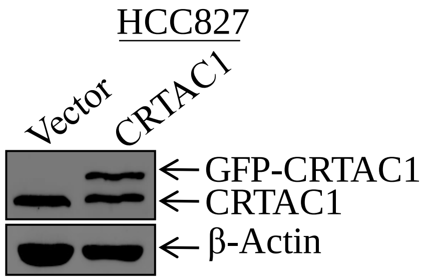

HCC827-β-actin

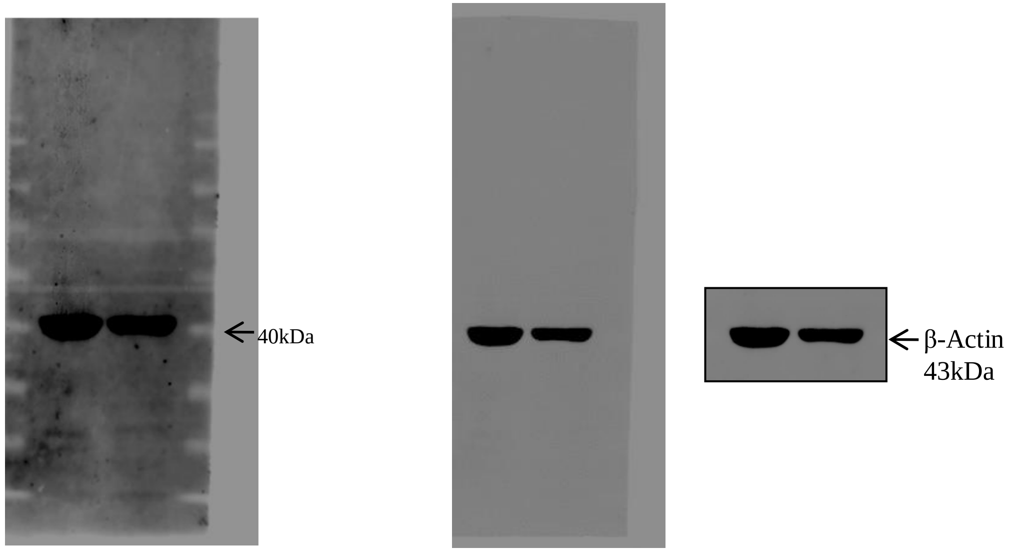

Figure2A

H226-CRTAC1

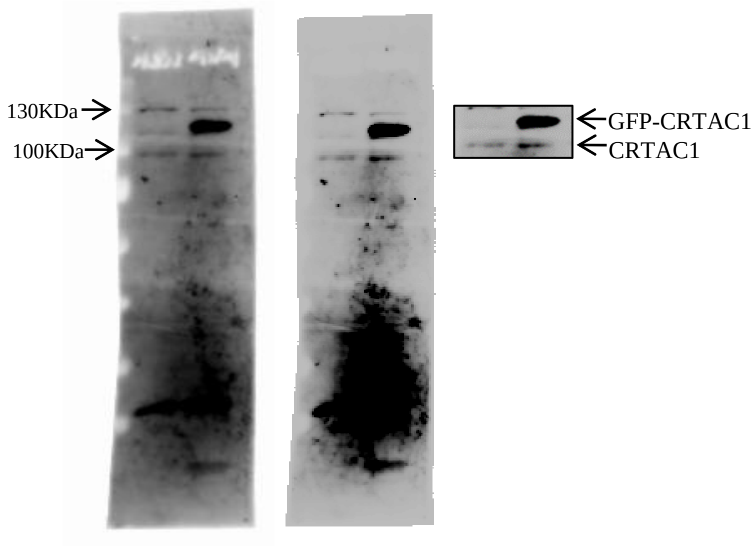

20210822-M2-226WZ-ACTIN-8.25-ST[FITC]

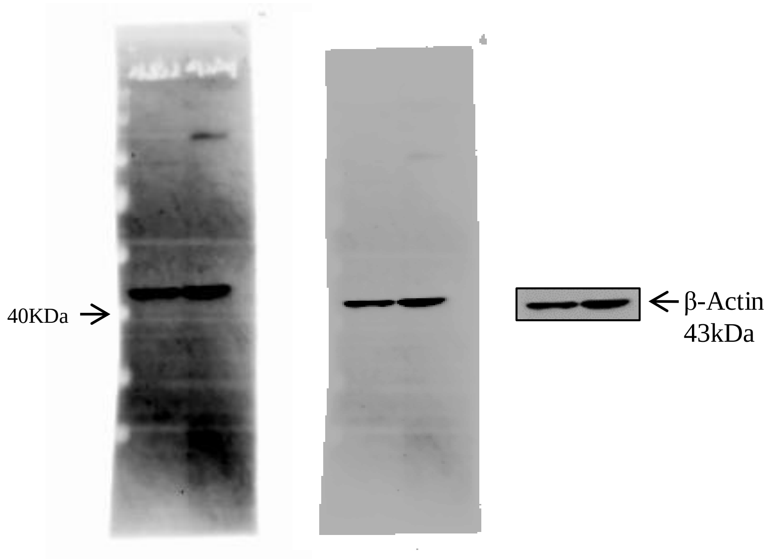

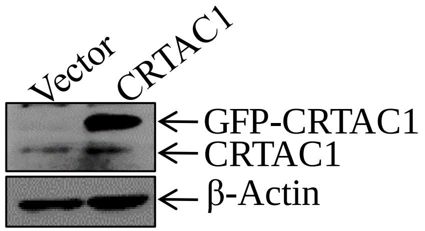

Figure2K

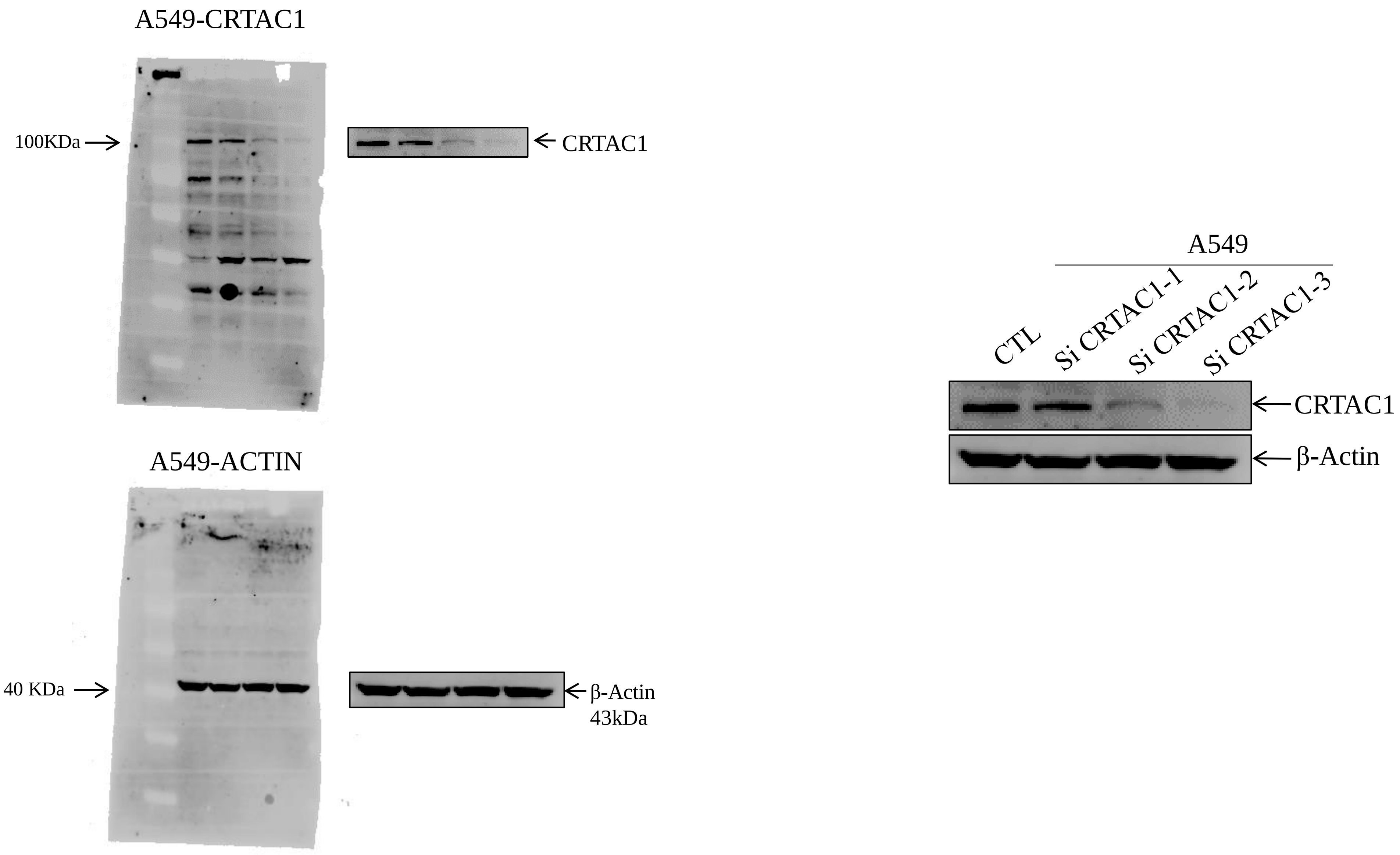
A549-CRTAC1
100KDa
CRTAC1
A549-ACTIN
40 KDa
β-Actin
43kDa
A549
CTL
Si CRTAC1-1
Si CRTAC1-2
Si CRTAC1-3
CRTAC1
β-Actin

Figure2K

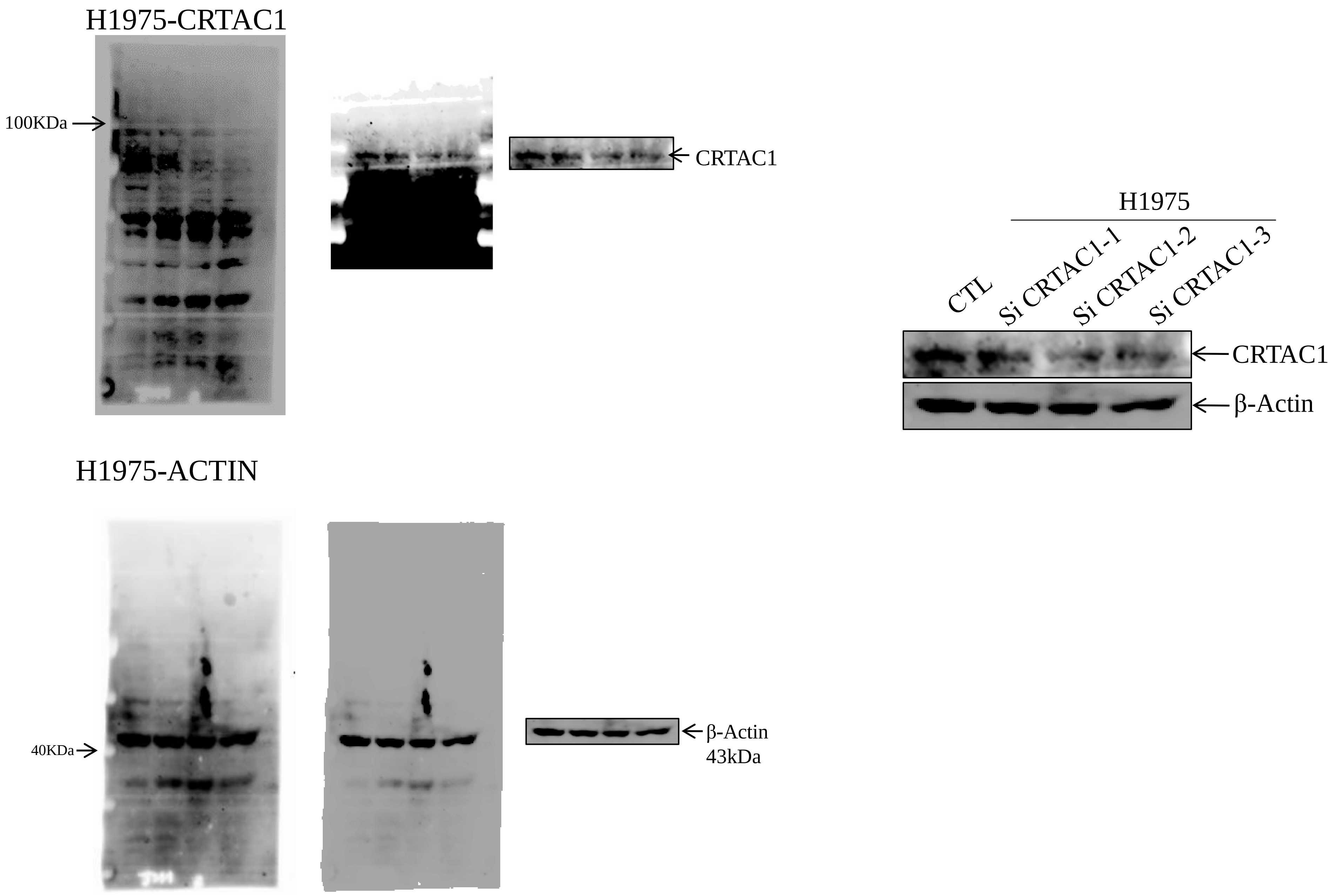
H1975-CRTAC1
100KDa
CRTAC1
H1975
CTL
Si CRTAC1-1
Si CRTAC1-2
Si CRTAC1-3
CRTAC1
β-Actin
H1975-ACTIN
40KDa
β-Actin
43kDa

Figure4A

H1299-Cleaved-Caspase3

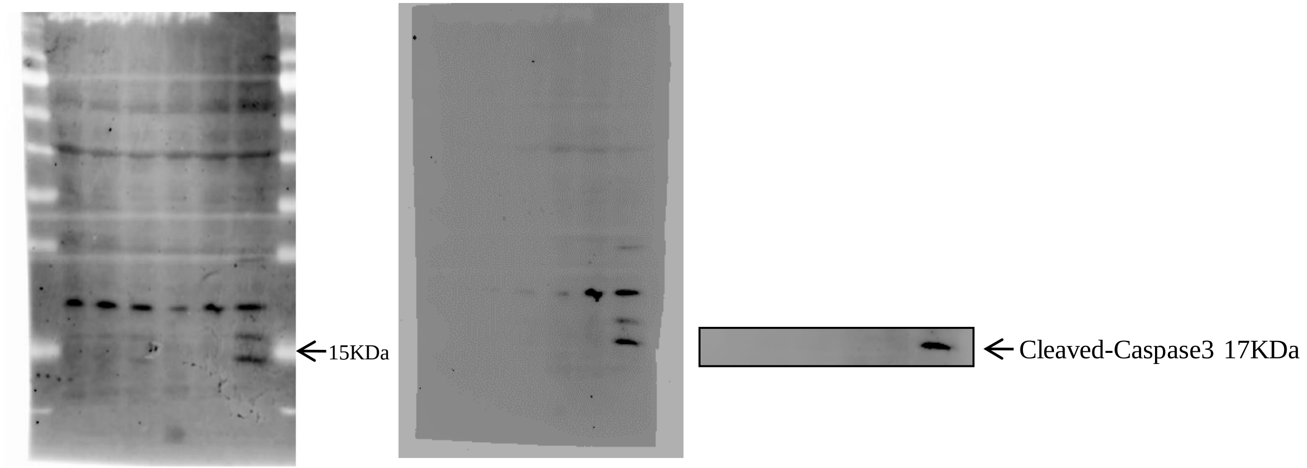

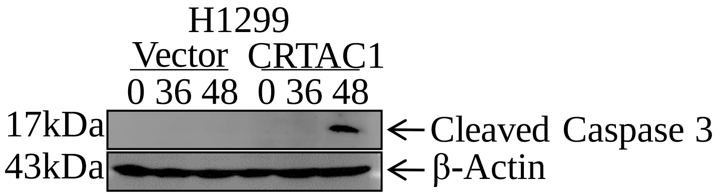

H1299-ACTIN

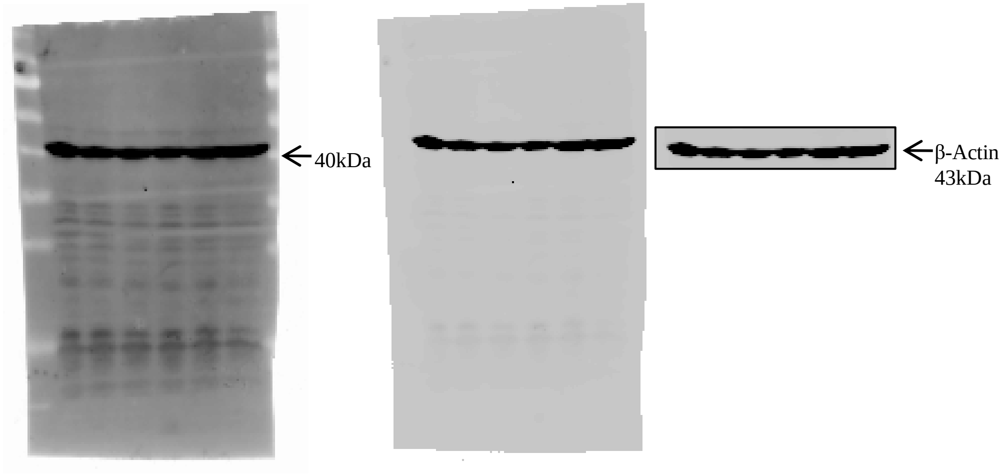

Figure4A

H1299-Cleaved-Caspase3

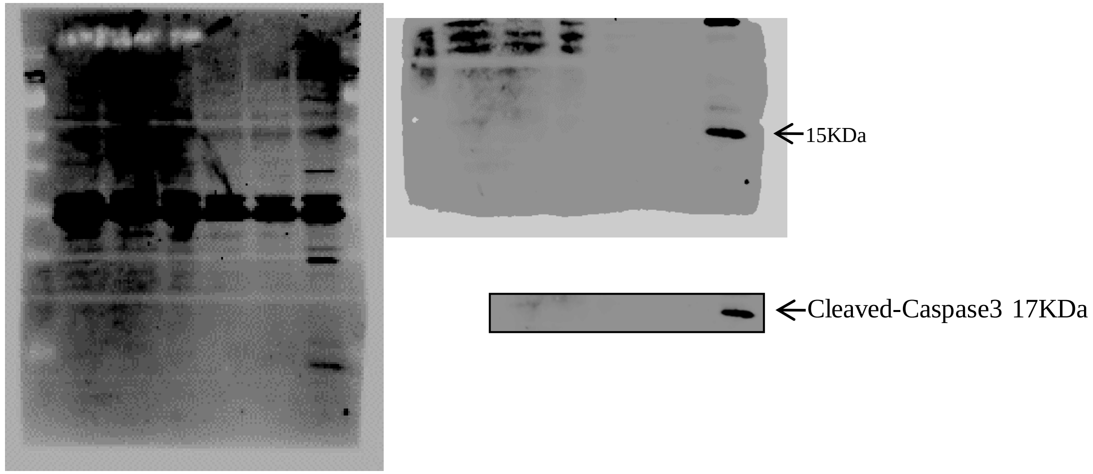

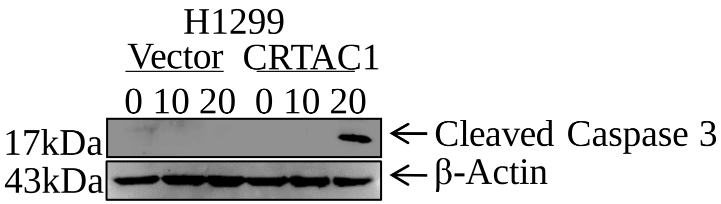

H1299-ACTIN

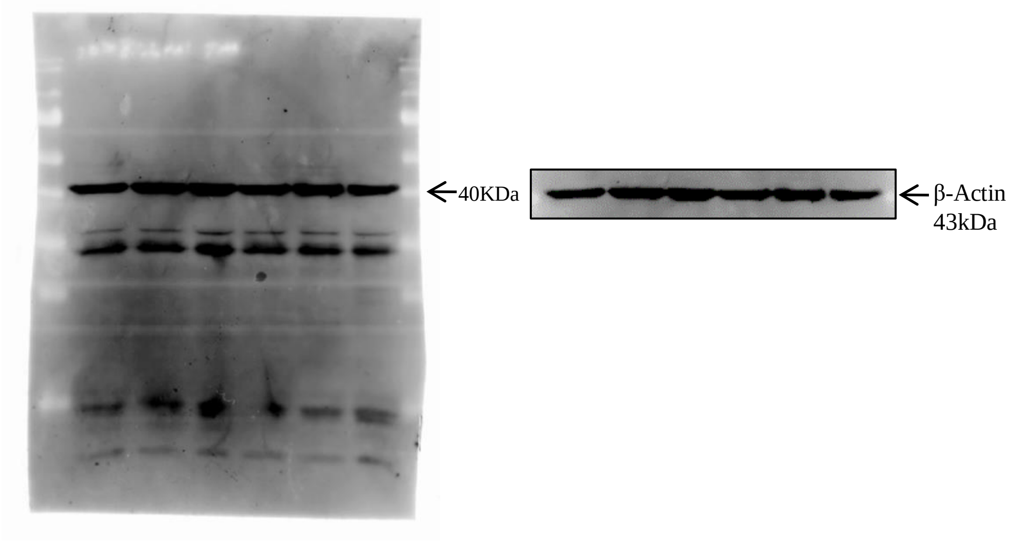

Figure4A

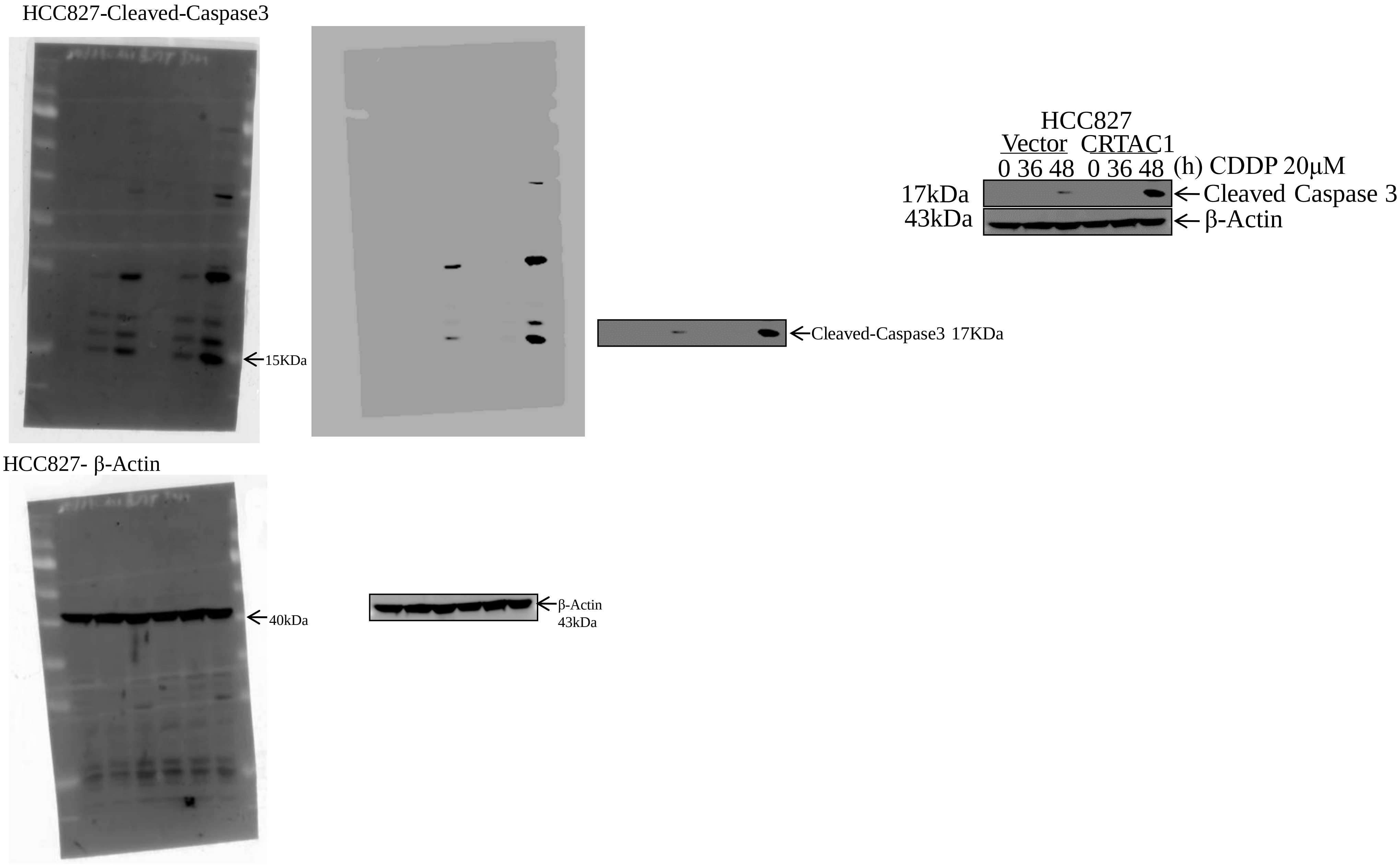
HCC827-Cleaved-Caspase3
15KDa
Cleaved-Caspase3 17KDa
HCC827
Vector
CRTAC1
0 36 48 0 36 48 (h) CDDP 20μM
17kDa
43kDa
Cleaved Caspase 3
β-Actin
HCC827- β-Actin
40kDa
β-Actin
43kDa

Figure4A

HCC827-Cleaved-Caspase3

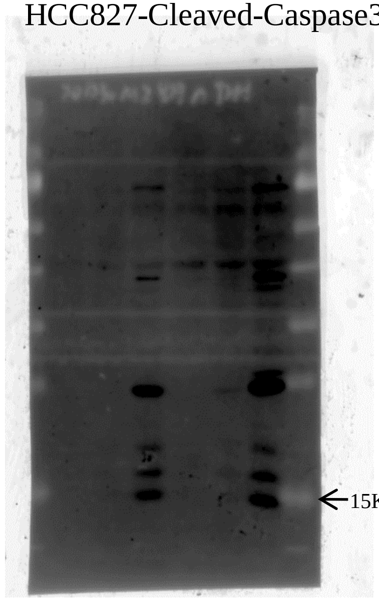

15KDa

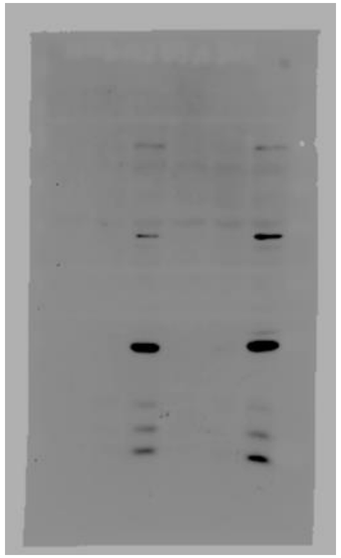

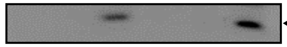

Cleaved-Caspase3 17KDa

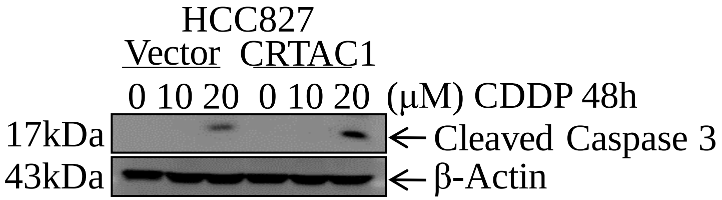

HCC827-ACTIN

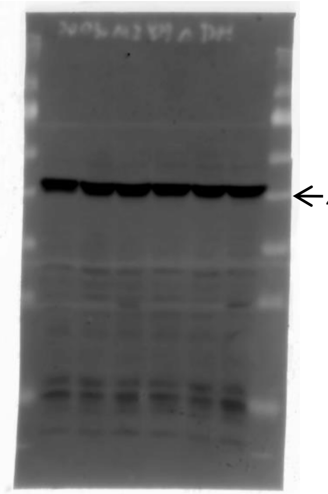

40kDa

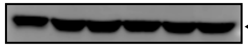

β-Actin
43kDa

Figure4C

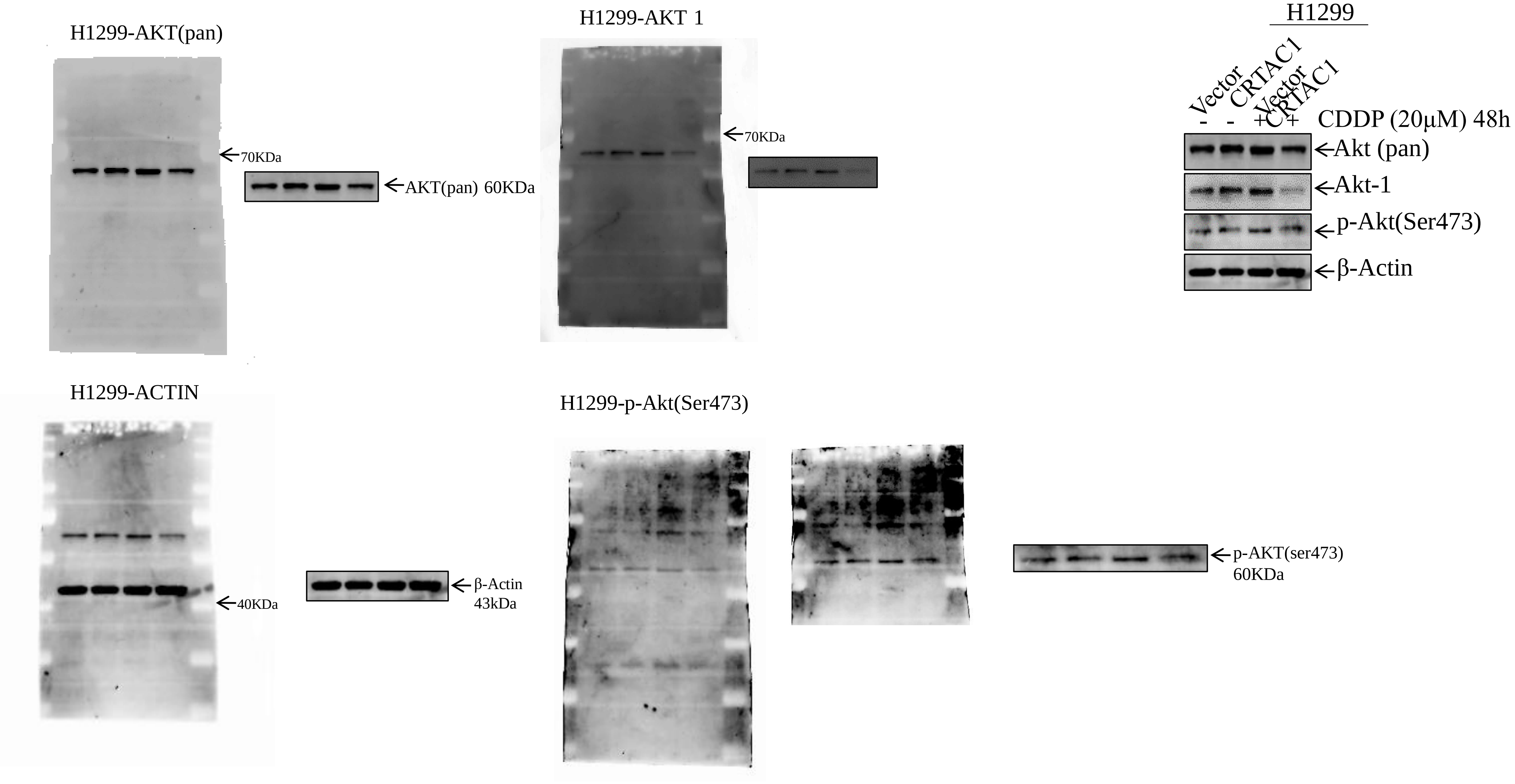
H1299-AKT(pan)
70KDa
AKT(pan) 60KDa
H1299-AKT 1
70KDa
H1299
Vector
CRTAC1
Vector
CRTAC1
- - + +
CDDP (20μM) 48h
Akt (pan)
Akt-1
p-Akt(Ser473)
β-Actin
H1299-ACTIN
40KDa
β-Actin
43kDa
H1299-p-Akt(Ser473)
p-AKT(ser473)
60KDa

Figure4C

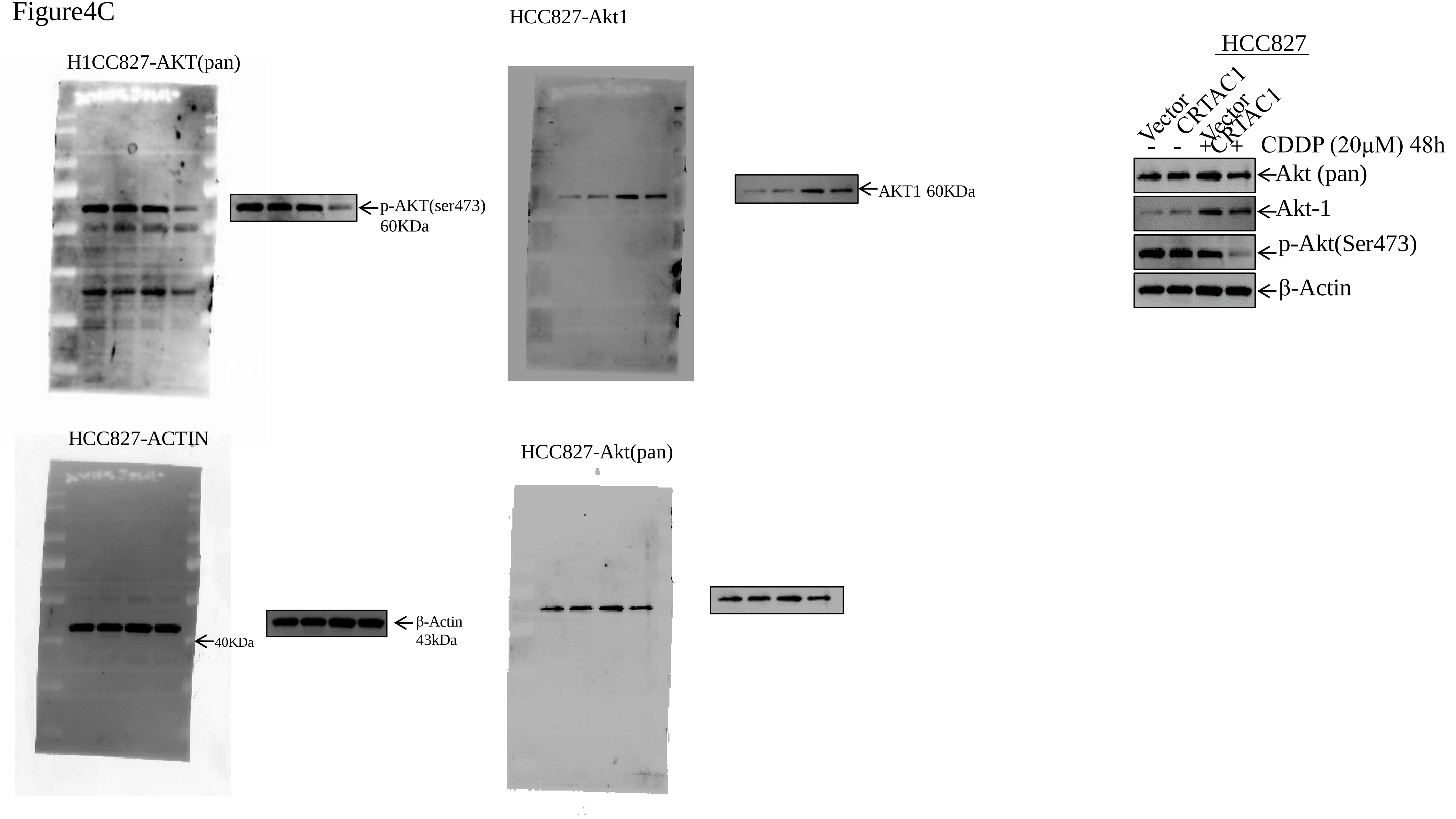
H1CC827-AKT(pan)
p-AKT(ser473)
60KDa
HCC827-Akt1
AKT1 60KDa
HCC827
Vector
CRTAC1
Vector
CRTAC1
- - + +
CDDP (20μM) 48h
Akt (pan)
Akt-1
p-Akt(Ser473)
β-Actin
HCC827-ACTIN
40KDa
β-Actin
43kDa
HCC827-Akt(pan)

Figure4C

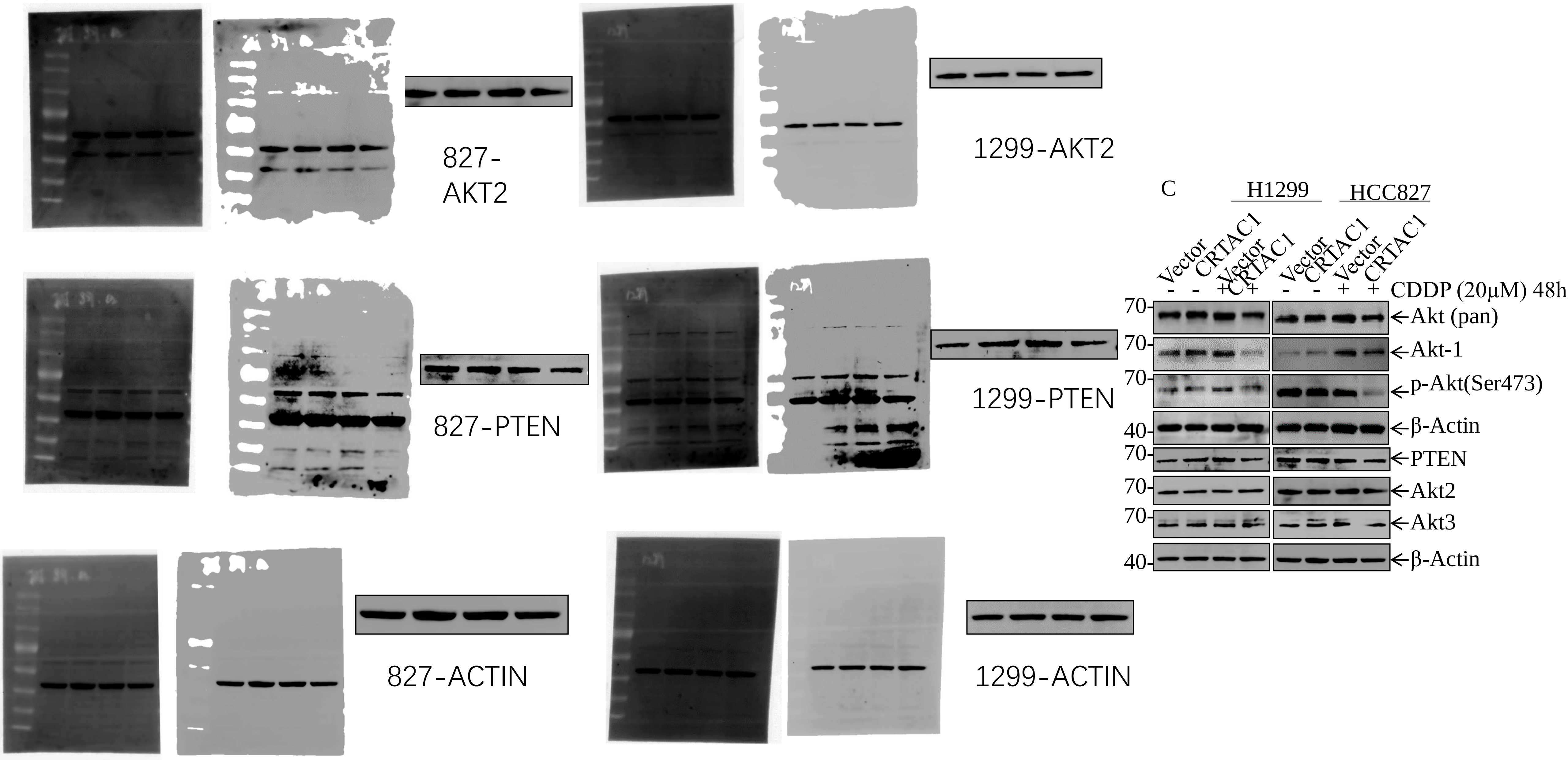
827-
AKT2
1299-AKT2
827-PTEN
1299-PTEN
827-ACTIN
1299-ACTIN
C
H1299
HCC827
Vector
CRTAC1
Vector
CRTAC1
Vector
CRTAC1
Vector
CRTAC1
CDDP (20μM) 48h
Akt (pan)
Akt-1
p-Akt(Ser473)
β-Actin
PTEN
Akt2
Akt3
β-Actin
70
70
70
40
70
70
70
40

Figure4C

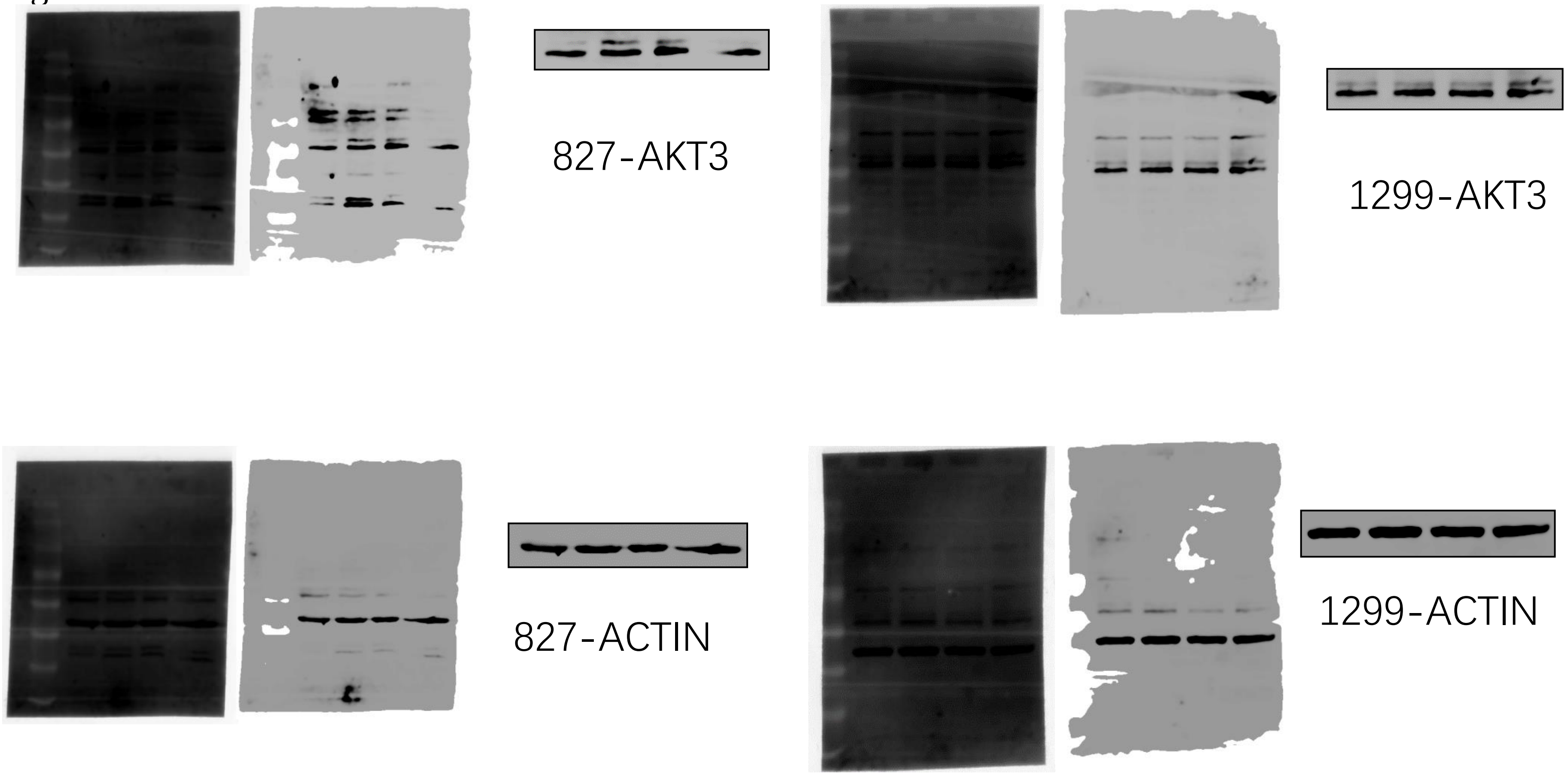

Figure5C

H1299-AKT1

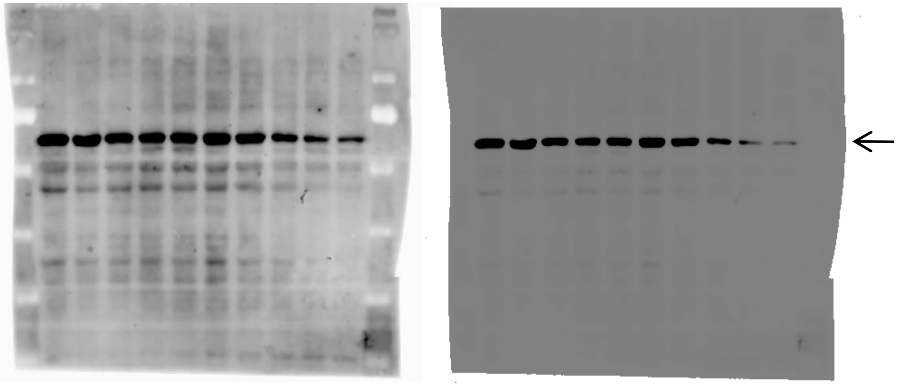

AKT1
60kDa

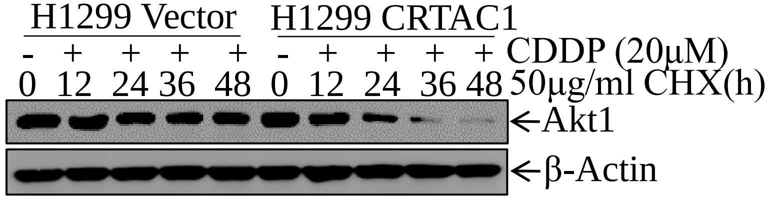

H1299-ACTIN

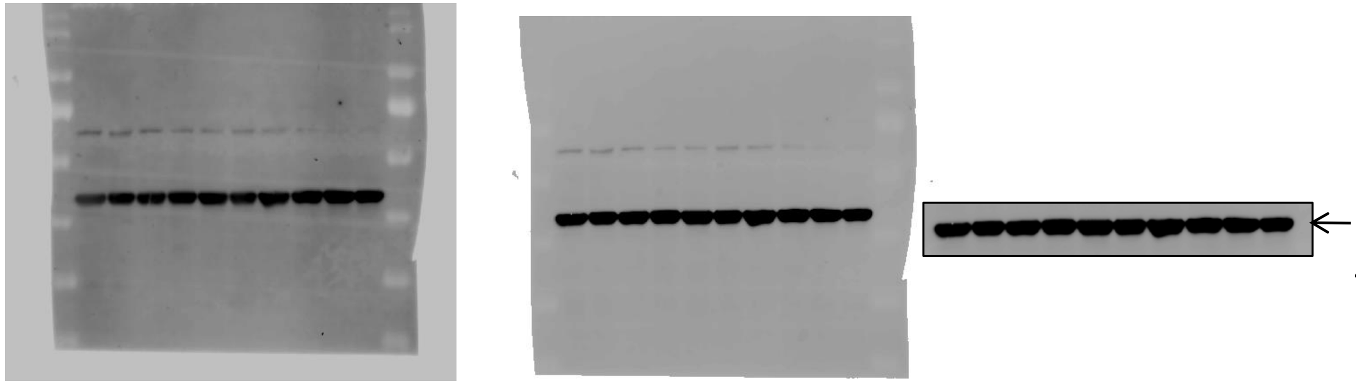

β-Actin
43kDa

Figure5D

H1299-Akt1

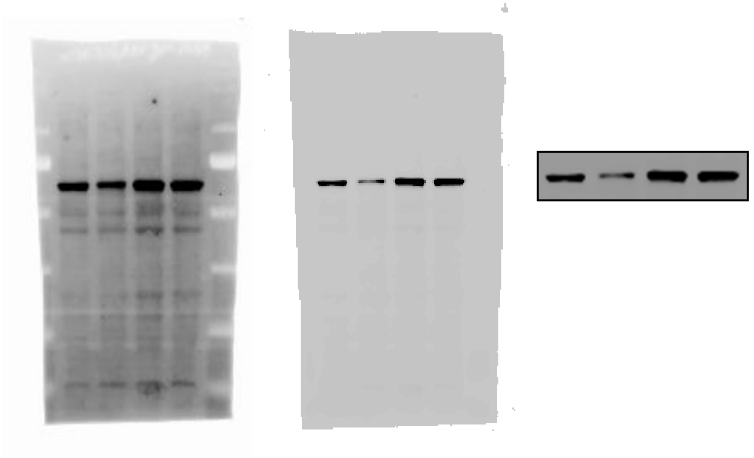

H1299-ACTIN

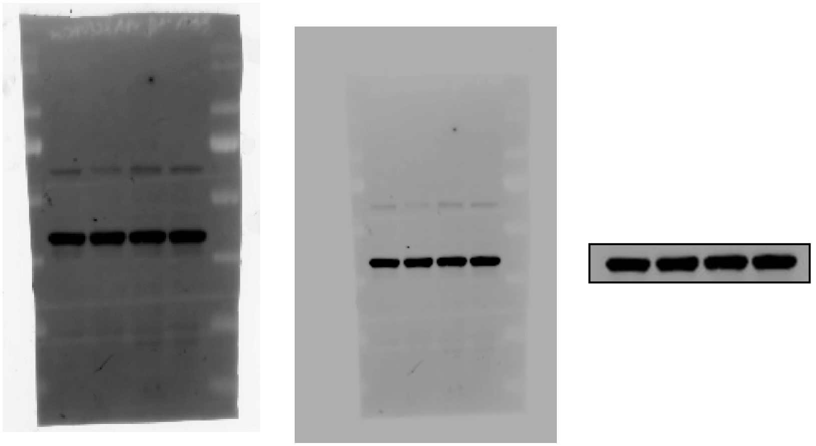

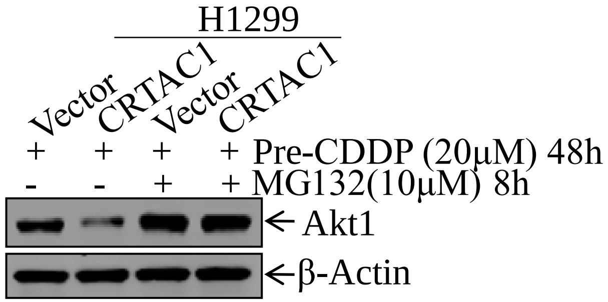

Figure5E

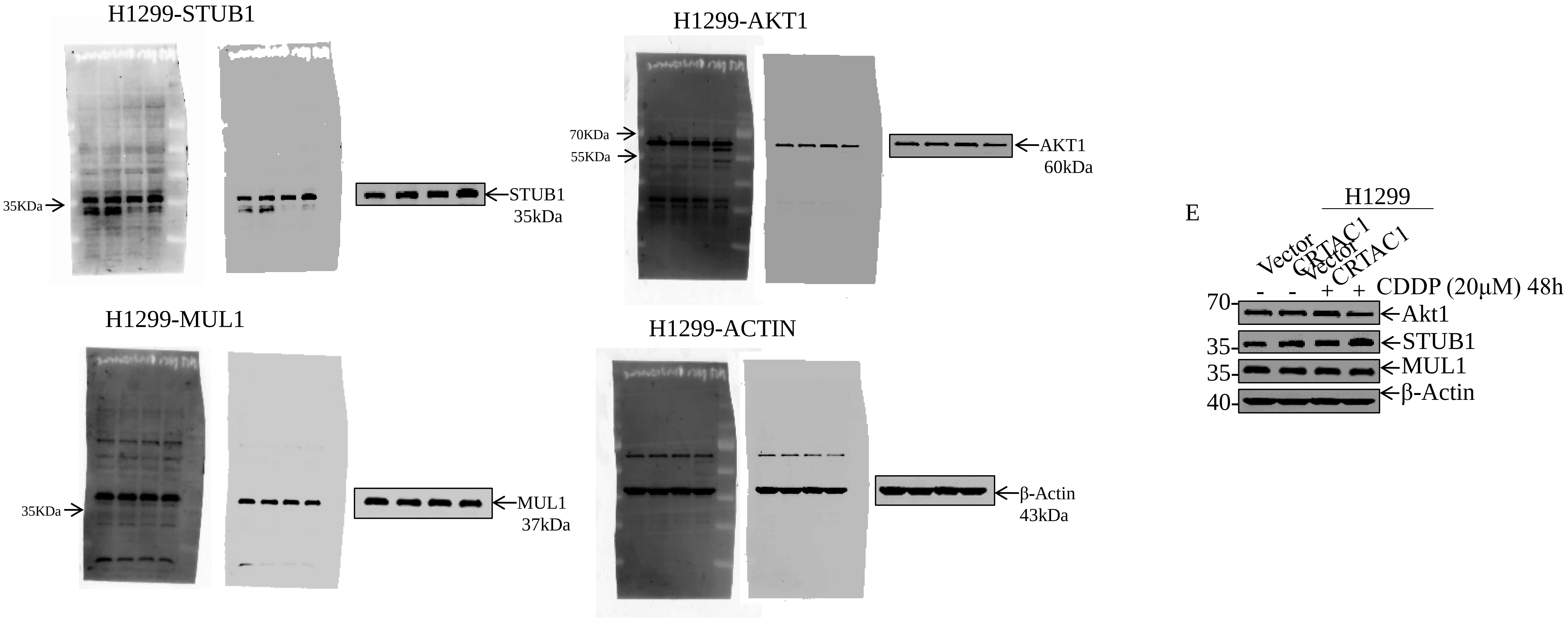
H1299-STUB1
35KDa
STUB1
35kDa
H1299-AKT1
70KDa
55KDa
AKT1
60kDa
H1299-MUL1
35KDa
MUL1
37kDa
H1299-ACTIN
β-Actin
43kDa
E
H1299
Vector
CRTAC1
Vector
CRTAC1
CDDP (20μM) 48h
- - + +
70
Akt1
35
STUB1
35
MUL1
40
β-Actin

Figure5E

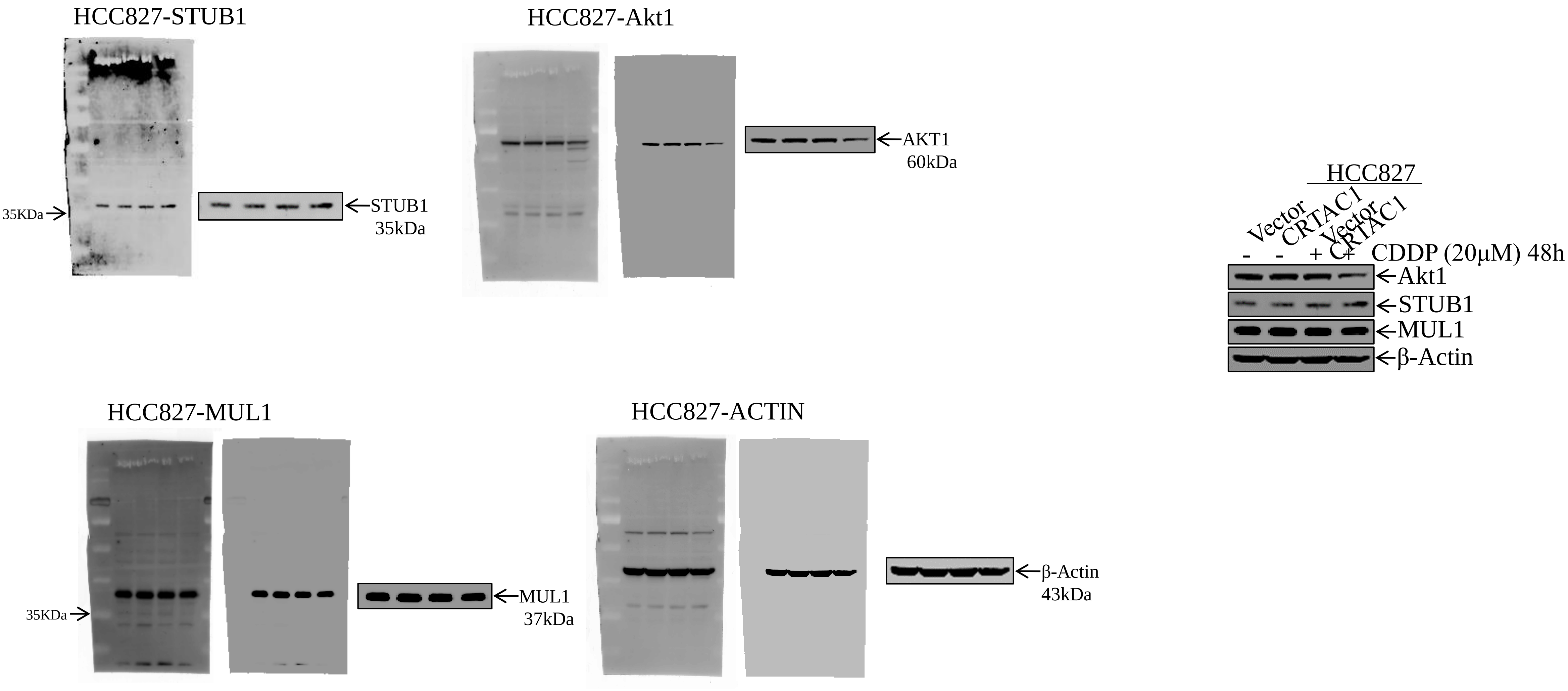

HCC827-STUB1
35KDa
STUB1
35kDa
HCC827-Akt1
AKT1
60kDa
HCC827
Vector
CRTAC1
Vector
CRTAC1
- - + +
CDDP (20μM) 48h
Akt1
STUB1
MUL1
β-Actin
HCC827-MUL1
35KDa
MUL1
37kDa
HCC827-ACTIN
β-Actin
43kDa

Figure5F

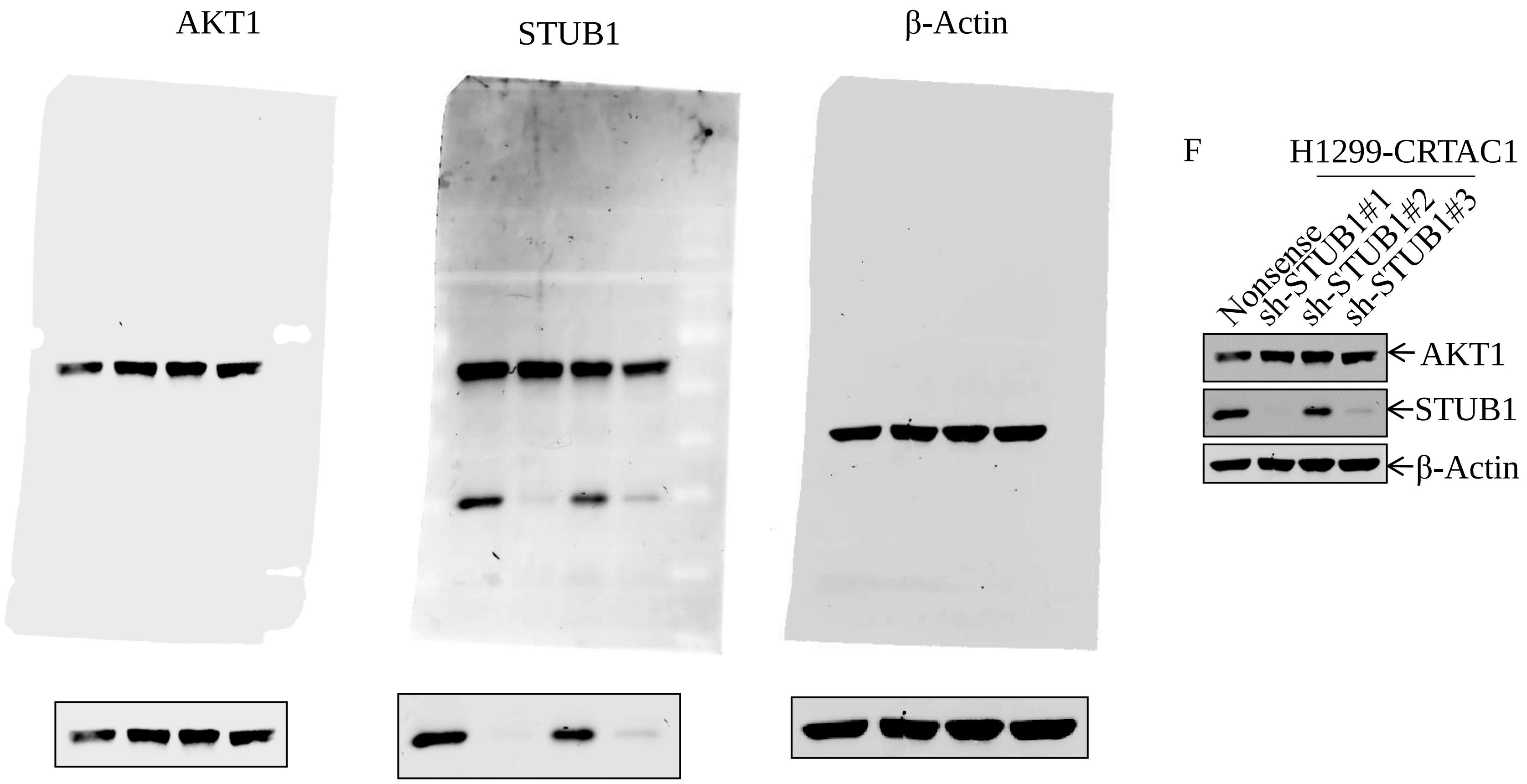
AKT1
STUB1
β-Actin
F
H1299-CRTAC1
Nonsense
sh-STUB1#1
sh-STUB1#2
sh-STUB1#3
AKT1
STUB1
β-Actin

Figure5J

Akt1

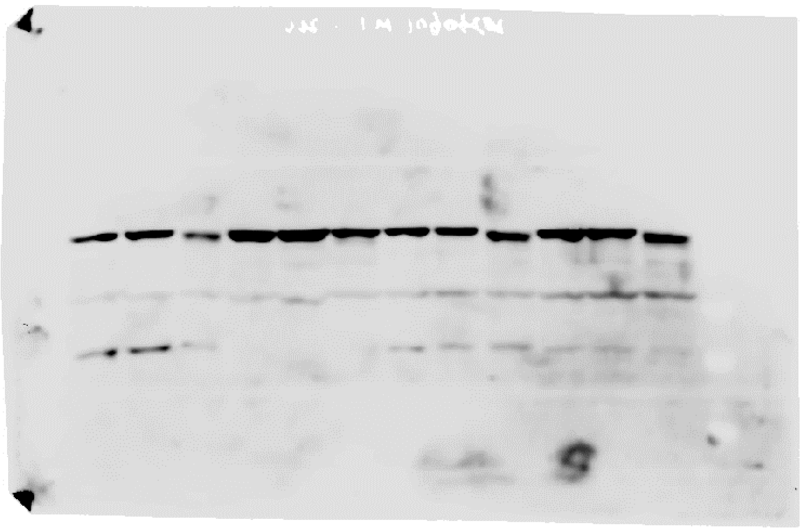

β-Actin

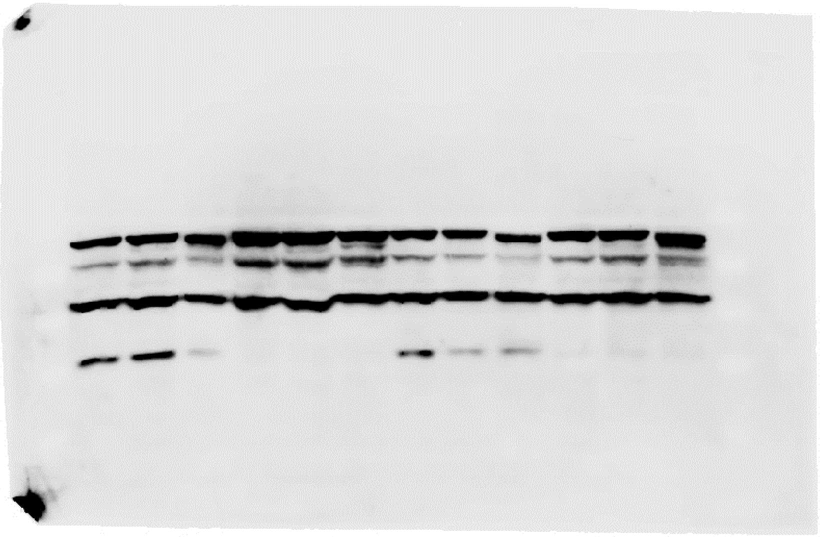

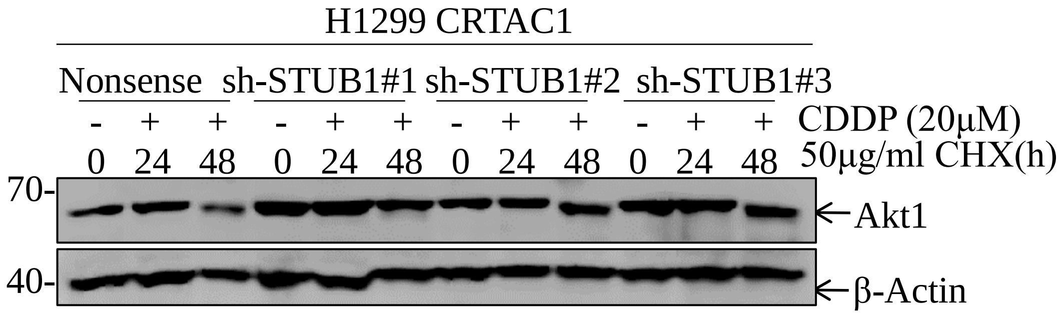

Figure6J

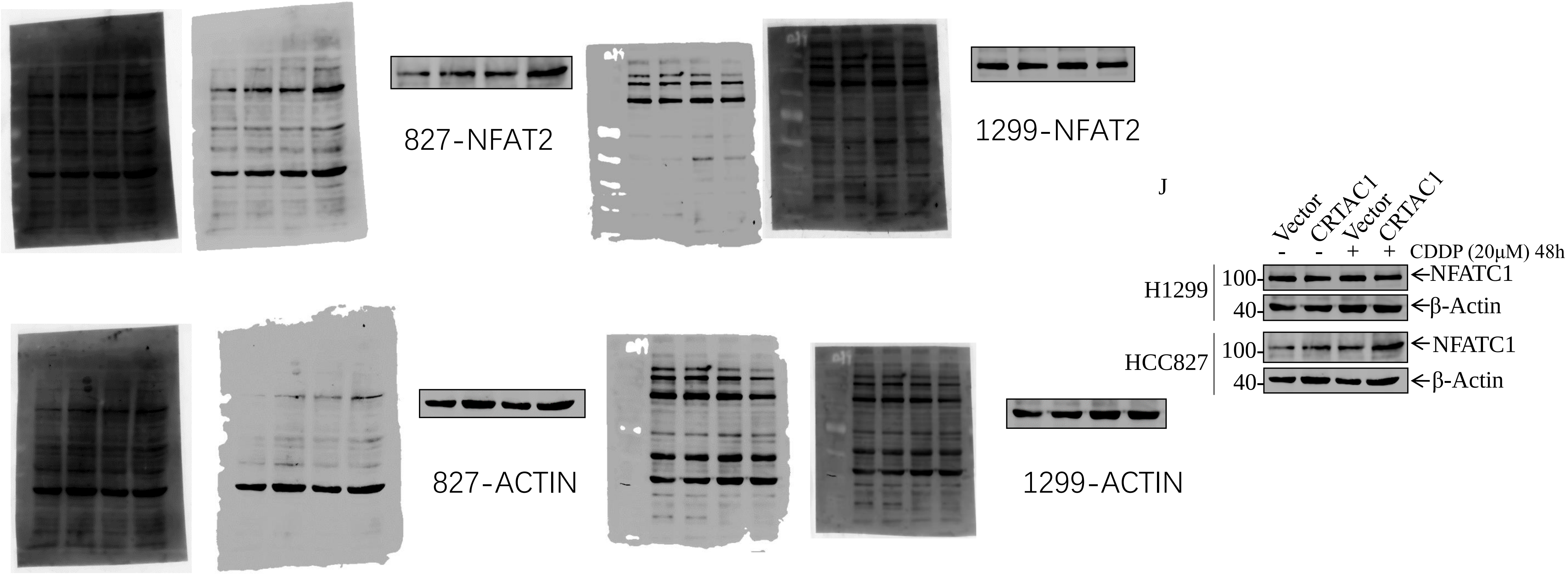
827-NFAT2
1299-NFAT2
827-ACTIN
1299-ACTIN
J
Vector
CRTAC1
Vector
CRTAC1
- - + + CDDP (20μM) 48h
H1299
100-
←NFATC1
40-
←β-Actin
HCC827
100-
←NFATC1
40-
←β-Actin

Figure6K

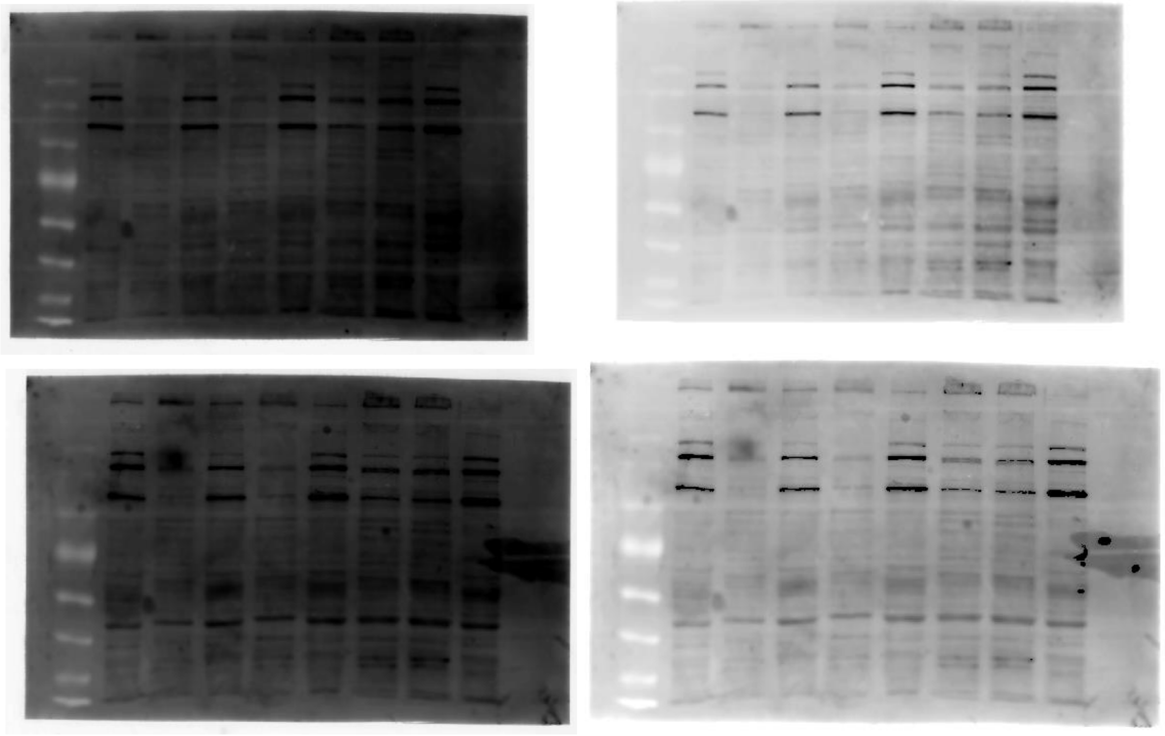

H1299-NFAT2

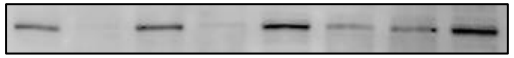

H1299-ACTIN

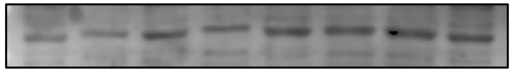

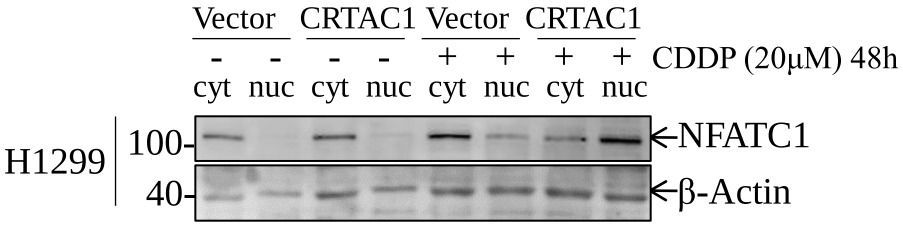

Figure6K

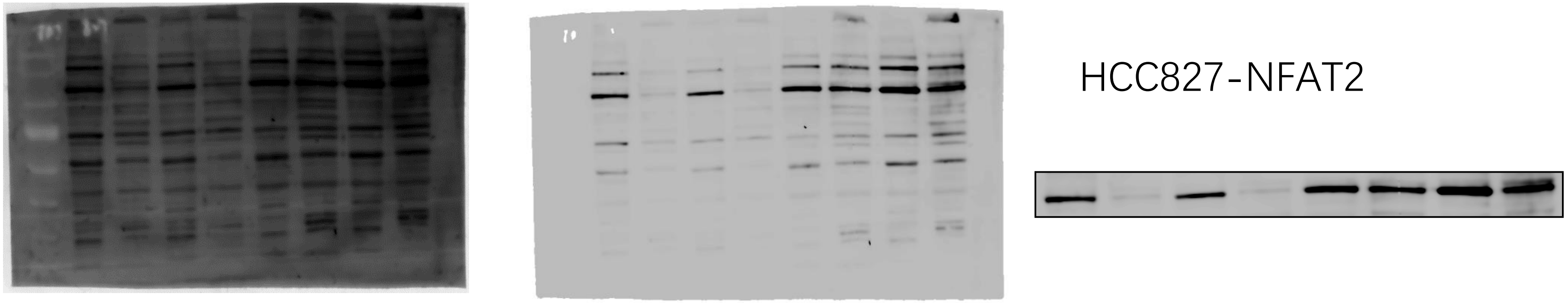

HCC827-NFAT2

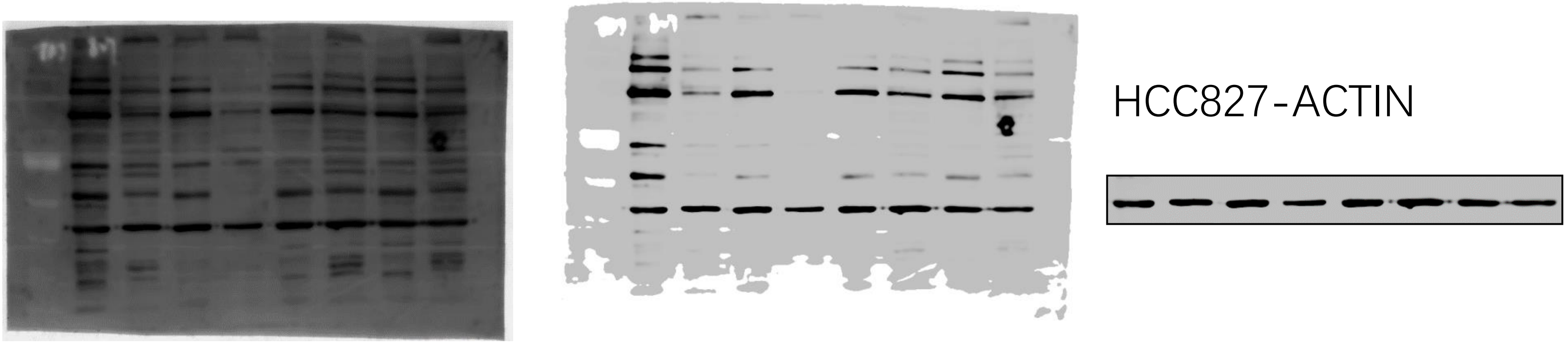

HCC827-ACTIN

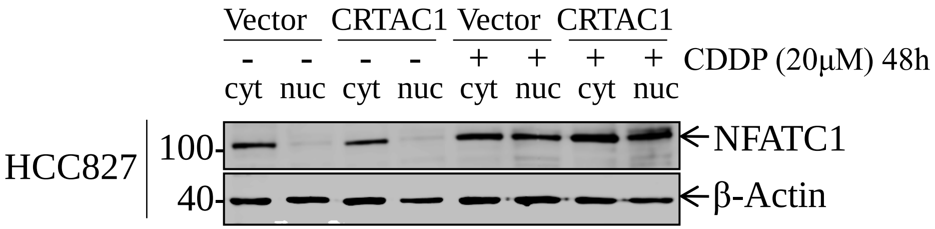

Vector
CRTAC1
Vector
CRTAC1
- - - - + + + +
CDDP (20μM) 48h
cyt nuc cyt nuc cyt nuc cyt nuc
HCC827
100-
40-
NFATC1
β-Actin

Figure7E

H1299-STUB1

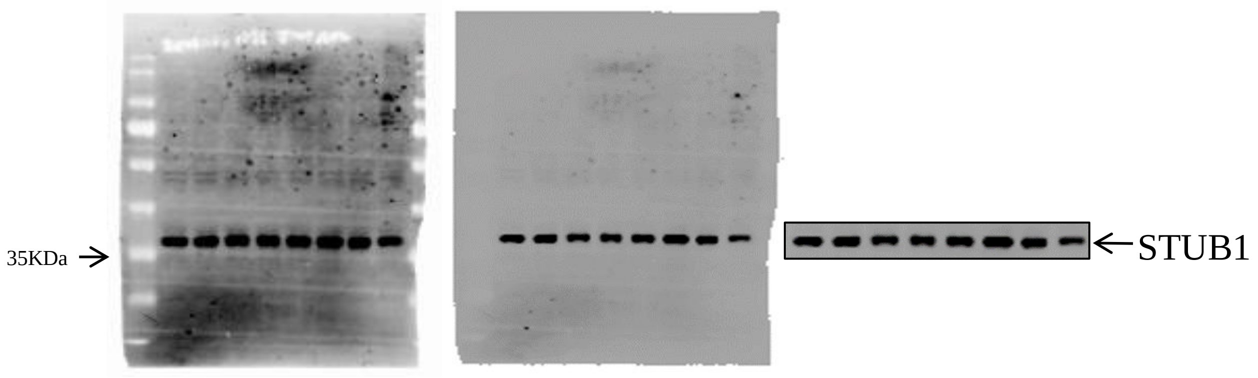

H1299-ACTIN

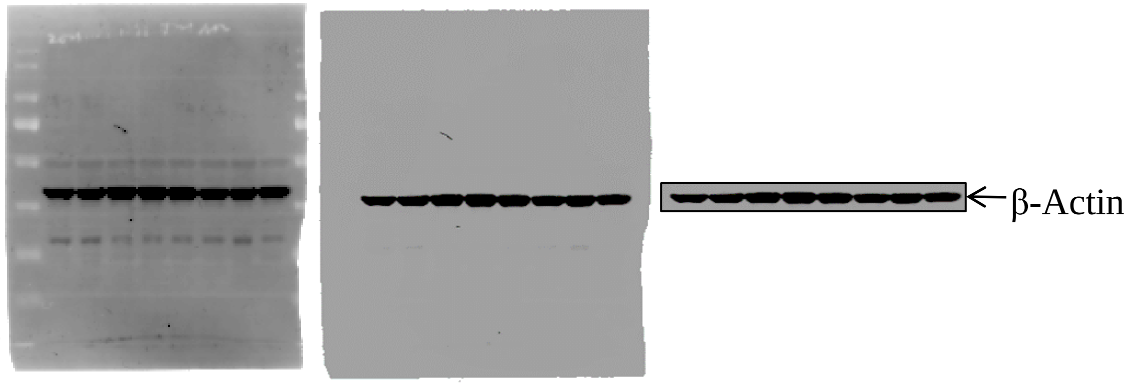

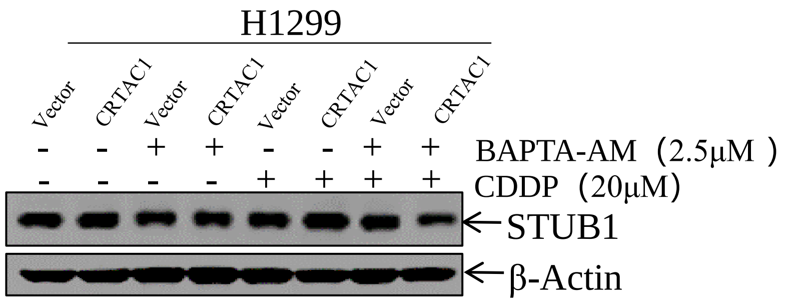

Figur7F

HCC827-STUB1

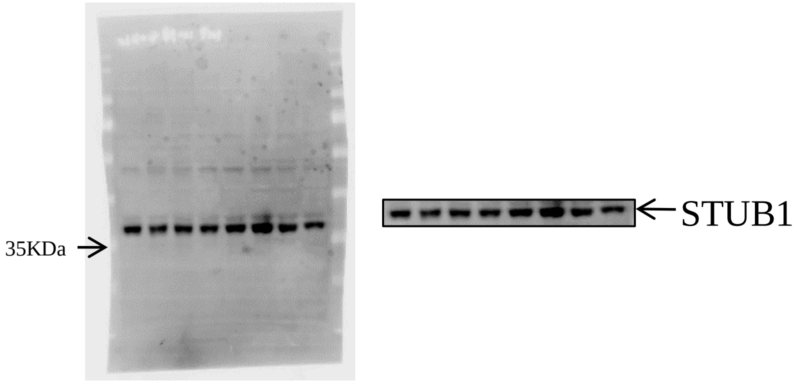

HCC827-ACTIN

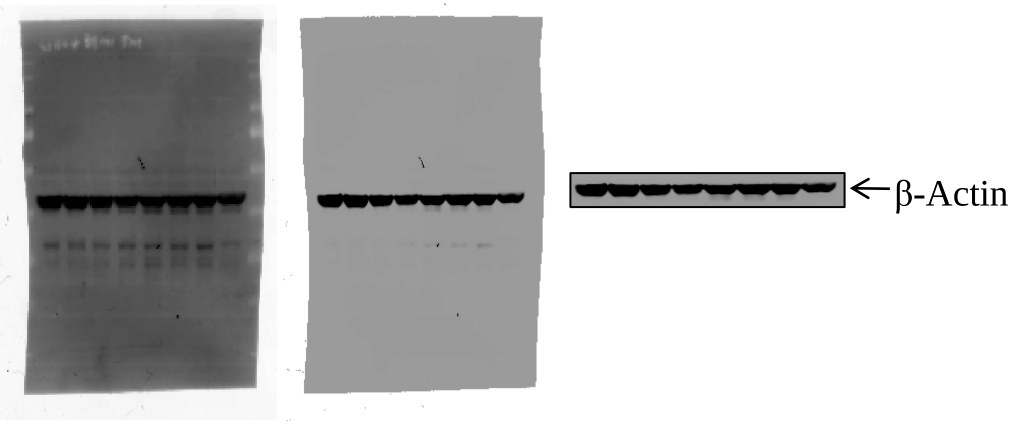

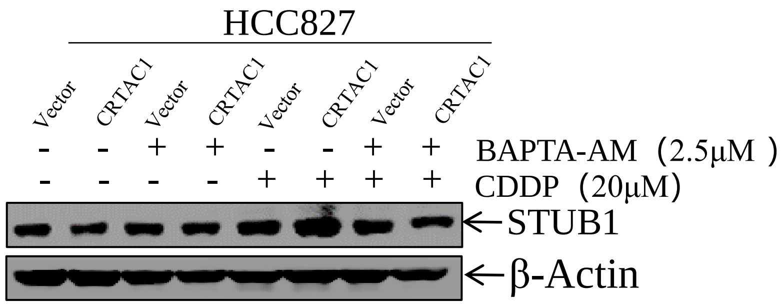

Supplement: Supplementary file 1 — Supplymentary Figure S1 [file 41419_2023_6088_MOESM1_ESM.pdf]
