# Supplementary material for: CRTAC1 enhances the chemosensitivity of non-small cell lung cancer to cisplatin by eliciting RyR-mediated calcium release and inhibiting Akt1 expression
Source: Cell Death Dis. 2023 Aug 26;14(8):563. doi: 10.1038/s41419-023-06088-1 (PMC10460435; doi:10.1038/s41419-023-06088-1)
Supplement: Supplementary file 2 — Supplemental Material_Original Western blots [file 41419_2023_6088_MOESM2_ESM.docx]

**Figure S1.** Akt inhibitor A-44365 increase the cisplatin chemotherapy sensitivity and cisplatin-induced apoptosis. (A, D) The effect of Akt inhibitor A-44365 on the cell viability of cisplatin-treated H1299 and HCC827 cells were analyzed using the ATP assay. (B, E) NSCLC cells were treated with A-44365 (200 nM), CDDP(20µM) alone or combined for 48 h, and apoptosis was detected by flow cytometry. (C, F) Statistical analysis of apoptosis in H1299/HCC827 cells treated with A-44365 and cisplatin. (*) indicates a significant difference (*P*< 0.05).
